# Selectivity analysis of diaminopyrimidine-based inhibitors of MTHFD1, MTHFD2 and MTHFD2L

**DOI:** 10.1038/s41598-024-71879-1

**Published:** 2024-09-10

**Authors:** Vibhu Jha, Leif A. Eriksson

**Affiliations:** 1 Department of Chemistry and Molecular Biology, University of Gothenburg, 405 30 Göteborg, Sweden; 2 School of Pharmacy and Medical Sciences, Faculty of Life Sciences, Institute of Cancer Therapeutics, University of Bradford, Bradford, BD71DP UK

**Keywords:** Diaminopyrimidine-based inhibitors, Molecular dynamics simulation, Molecular docking, Substrate binding site, Conformational changes, Molecular dynamics, Computational chemistry, Drug discovery and development, Structure-based drug design, Drug development

## Abstract

The mitochondrial enzyme methylenetetrahydrofolate dehydrogenase (MTHFD2) is involved in purine and thymidine synthesis via 1C metabolism. MTHFD2 is exclusively overexpressed in cancer cells but absent in most healthy adult human tissues. However, the two close homologs of MTHFD2 known as MTHFD1 and MTHFD2L are expressed in healthy adult human tissues and share a great structural resemblance to MTHFD2 with 54% and 89% sequence similarity, respectively. It is therefore notably challenging to find selective inhibitors of MTHFD2 due to the structural similarity, in particular protein binding site similarity with MTHFD1 and MTHFD2L. Tricyclic coumarin-based compounds (substrate site binders) and xanthine derivatives (allosteric site binders) are the only selective inhibitors of MTHFD2 reported till date. Nanomolar potent diaminopyrimidine-based inhibitors of MTHFD2 have been reported recently, however, they also demonstrate significant inhibitory activities against MTHFD1 and MTHFD2L. In this study, we have employed extensive computational modeling involving molecular docking and molecular dynamics simulations in order to investigate the binding modes and key interactions of diaminopyrimidine-based inhibitors at the substrate binding sites of MTHFD1, MTHFD2 and MTHFD2L, and compare with the tricyclic coumarin-based selective MTHFD2 inhibitor. The outcomes of our study provide significant insights into desirable and undesirable structural elements for rational structure-based design of new and selective inhibitors of MTHFD2 against cancer.

## Introduction

Methylenetetrahydrofolate dehydrogenase/cyclohydrolase (MTHFD2) is a bifunctional mitochondrial enzyme that plays a key role in 1C metabolism in purine and thymidine synthesis. MTHFD2 catalyzes the dehydrogenation of 5,10-methylene-THF (CH2–THF) with an NAD+ cofactor and cyclohydrolysis of 5,10-methenyl-THF (CH = THF), yielding 10-formyl-THF (CHO–THF), to ultimately produce formate as a 1C unit^1^. MTHFD2 has attracted significant interest as overexpressed levels thereof were detected in various tumors such as breast cancer^2^, colorectal cancer^3^, acute myeloid leukaemia^4,5^, renal cell carcinoma^6^ and hepatocellular carcinoma^7^. Increased risk of bladder cancer is also associated with upregulation of MTHFD2^8^. Reduction in MTHFD2 level has reportedly demonstrated tumor suppression effects in the aforementioned cancer types^4,9,10^. Inhibition of MTHFD2 potentiates increased oxidative stress^3^, glycine dependency^11^, and inadequate purine synthesis^4,9^ within the tumor cells, and furthermore suppresses mTORC1 activity through multiple mechanisms including guanine depletion and subsequent inhibition of the mTORC1-activating GTPase Rheb^12,13^. Shi et al.^14^ investigated the expression of MTHFD2 in The Cancer Genome Atlas (TCGA) Lung Adenocarcinoma (LUAD) samples and found that MTHFD2 was expressed higher in male patients than female patients and was positively associated with smoking habit, smoking years, and individual cancer stage^14^. MTHFD2 is hypothesized to effectuate non-metabolic functions involving RNA processing and epigenetic modification^15,16^, which are required for cancer cell proliferation^17^, and is considered a hot target for cancer drug discovery due to its absence or mild expression in healthy adult human tissues. Thus, MTHFD2-overpexpressing cancers can be targeted by developing potent MTHFD2 inhibitors which presents a new promising therapeutic strategy with minimal side effects^18^. However, the two close homologs of MTHFD2: MTHFD1 and MTHFD2L are expressed in healthy adult human tissues as well as in embryonic cells^19^. MTHFD1 shares 36% sequence identity and 54% sequence similarity with MTHFD2 whereas MTHFD2L shares significantly greater sequence identity (72%) and sequence similarity (89%) with MTHFD2^19^. The similarities of the proteins thus give rise to crucial selectivity issues that must be taken into account for the development of new MTHFD2 inhibitors.

**Fig. 1:**
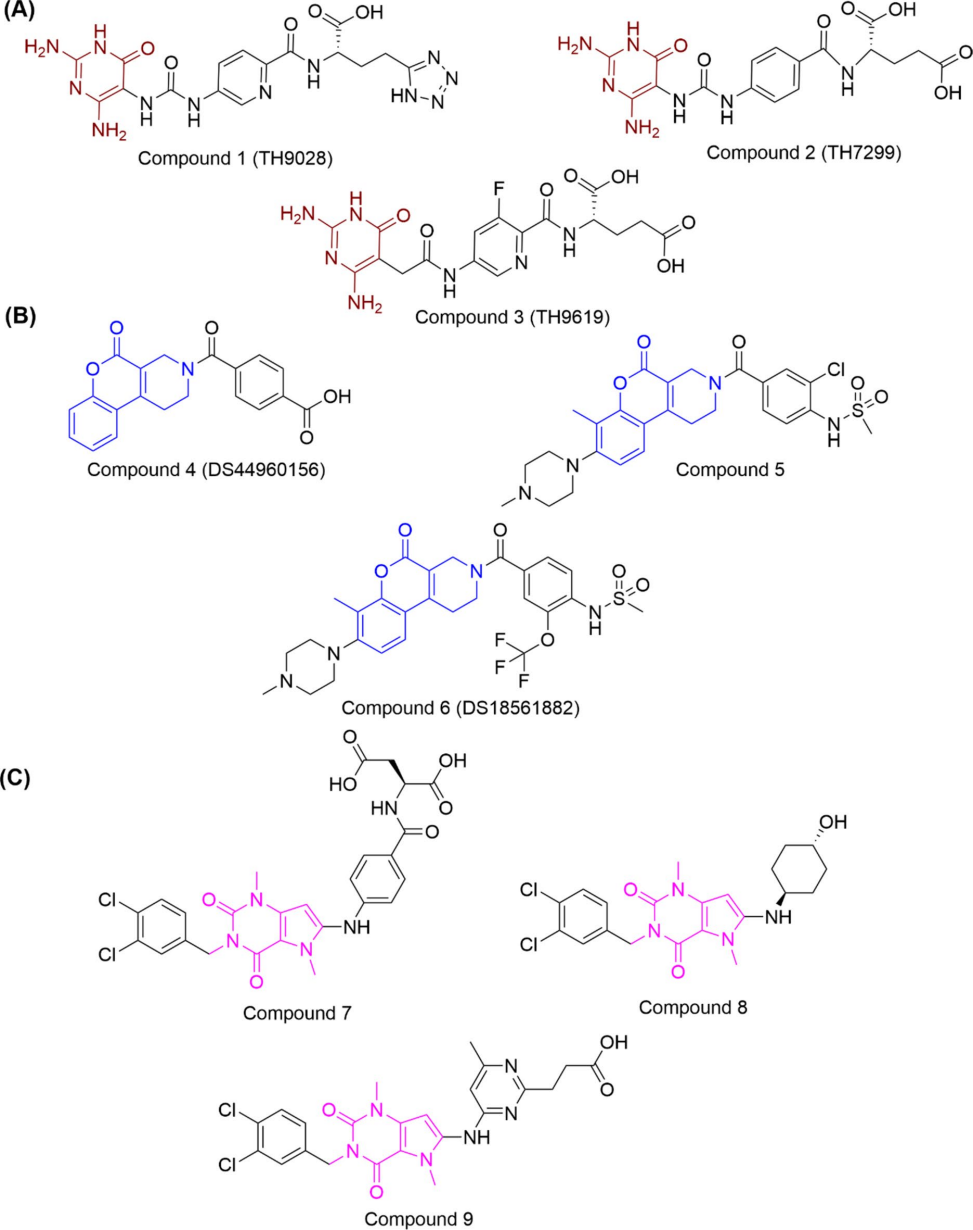
(**A**) Diaminopyrimidine-based non-selective inhibitors of MTHFD2 that bind to the substrate site (compounds **1–3**, studied in this work). (**B**) Tricyclic coumarin-based selective inhibitors of MTHFD2 that bind to the substrate binding site (compounds **4–6**). (**C**) Xanthine-based selective inhibitors of MTHFD2 that bind to the allosteric site (compound **7–9**).

Very recently, Bonagas et al.^5^ discovered a new series of diaminopyrimidine-based MTHFD2 inhibitors (Fig. 1A): TH9028 (compound **1**), TH7299 (compound **2**) and TH9619 (compound **3**), demonstrating IC_50_ values in the nanomolar range. The diaminopyrimidine-based compounds **1–3** were developed after a high throughput screening (HTS) campaign involving over 500,000 lead-like compounds, followed by structure-based optimization studies^5^. Compounds **1–3** were co-crystallized with MTHFD2 and shown to bind to the substrate binding site (PDB codes: 6S4A, 6S4E and 6S4F for compounds **1, 2** and **3**, respectively). Compound **1** was found to be the most potent MTHFD2 inhibitor of the series with an IC_50_ of 11 nM in biochemical assays, whereas compounds **2** and **3** exhibited IC_50_ values of 254 nM and 47 nM, respectively. The diaminopyrimidine-based inhibitors furthermore resulted in lowered replication fork, replication stress, S-phase arrest and subsequent apoptosis of acute myeloid leukaemia cells in vitro and in vivo. Moreover, compounds **1–3** hampered production of thymidine, leading to misincorporation of uracil into DNA and replication stress. Despite the encouraging results against MTHFD2 both in vitro and in vivo, the aforementioned diaminopyrimidine-based inhibitors also possess potent inhibition of both MTHFD1 and MTHFD2L isoforms which further confirms the non-selective mode of inhibition. Compound **1, 2** and **3** demonstrated IC_50_ values of 0.5 nM, 89 nM and 16 nM, respectively, against MTHFD1 in the biochemical screening, whereas for MTHFD2L the IC_50_ values were 27 nM, 126 nM and 47 nM, respectively^5^. The selectivity issues of the diaminopyrimidine-based inhibitors were furthermore addressed by the same research group in a different work^19^, emphasizing the potential challenges in developing selective MTHFD2 inhibitors aimed at no or poor inhibition of the MTHFD1 and MTHFD2L isoforms. In the past, a few dual inhibitors of MTHFD1/2 were also identified. A folate analogue named LY345899^1,18^ (Fig. S1) impeded tumor growth in a mice xenograft model of colorectal cancer through intraperitoneal injection^1^, however, demonstrated greater inhibitory activity against MTHFD1 (IC_50_: 96 nM) over MTHFD2 (IC_50_: 663 nM), thus presenting potential safety risk. Likewise, a natural product named carolacton (Fig. S1) reportedly binds to both the MTHFD1 and MTHFD2 isoforms, showing K_i_ values in the nanomolar range^20^. These potent compounds along with the aforementioned diaminopyrimidine-based inhibitors display concurrent inhibition of MTHFD1/2, and may thus be considered undesirable due to the high expression of MTHFD1 in healthy adult human tissues.

Kawai et al.^21,22^ developed a series of tricyclic coumarin-based selective MTHFD2 inhibitors (Fig. 1B) that bind to the substrate binding site. Lee et al.^23^ discovered xanthine-based derivatives (Fig. 1C) that bind selectively to the MTHFD2 isozyme by occupying the allosteric site. To the best of our knowledge, the tricyclic coumarin-based inhibitors and xanthine-based derivatives are the only selective inhibitors of MTHFD2 reported till date. The tricyclic coumarin-based inhibitor series (‘DS’ series) was developed from structure-guided optimization of their HTS hit (Fig. S1) which is a tetrahydropyrido[4,3-d]pyrimidin-4-one derivative and a substrate site binder of MTHFD2. The HTS hit (PDB code: 6JID) demonstrated moderate inhibition against MTHFD2 with an IC_50_ of 8.3 μM, however, encouragingly, possessed no inhibition against the MTHFD1 isoform, with IC_50_ > 100 μM^21^. The first tricyclic coumarin-based lead inhibitor DS44960156 (compound **4**, PDB code: 6JIB, Fig. 1B) showed an IC_50_ of 1.6 µM in enzyme assays, possessing > 18-fold selectivity for MTHFD2. The substitution of the central tetrahydropyrido-pyrimidin-4-one ring (HTS hit) with the tricyclic coumarin-based ring (compound 4) was an integral factor in significantly improving MTHFD2 inhibition. Furthermore, structure-guided optimization studies led to the discovery of compound **5** (PDB code: 6KG2, Fig. 1B), showing IC_50_ values of 0.048 µM and 6.4 µM for MTHFD2 and MTHFD1, respectively, possessing > 133-fold selectivity for MTHFD2. DS18561882 (compound **6**, Fig. 1B) was identified as the best of the ‘DS’ series demonstrating 0.0063 µM and 0.57 µM IC_50_ values for MTHFD2 and MTHFD1 respectively, with > 250-fold selectivity for MTHFD2. Moreover, compound **6** was developed as an orally available MTHFD2 inhibitor with a decent pharmacokinetic profile and high cell-based activity of 0.14 µM GI_50_ against the MDA-MB-231 cell line derived from human breast cancer^22^.

The xanthine-based derivatives by Lee et al.^23^ occupy an allosteric site of MTHFD2 and thus obstruct the binding of the cofactor and phosphate to the MTHFD2 enzyme. The three allosteric inhibitors (compounds **7–9**, Fig. 1C) were co-crystallized with MTHFD2 (PDB codes: 7EHM, 7EHV and 7EHN for compounds **7, 8** and **9**, respectively), all of which coexist with a folate-based inhibitor (compound **10**, Fig. S1). The co-crystallized poses confirm that the xanthine-based derivatives were accommodated into the allosteric site of MTHFD2 whereas the folate-based compound **10** binds to the substrate binding site of MTHFD2. In addition, another X-ray crystal structure of MTHFD2 was solved, attributed by the absence of any allosteric inhibitor, however, with the presence of compound **10**, cofactor NAD^+^ and pyrophosphate (P_i_) (PDB code: 7EHJ). On comparing and superposing the aforementioned X-ray structures, it was found that MTHFD2 undergoes notable conformational changes at its allosteric site when the xanthine-based derivatives are bound, while in the absence of the xanthine-based derivatives, no conformational changes were observed. Kinetic studies furthermore confirmed the competitive mode of MTHFD2 inhibition by compound **10** while the xanthine-based derivatives illustrated non-competitive mode of enzyme inhibition. The overall findings of those studies corroborate a novel allosteric binding mode by the formation of catalytically inactive ternary complexes (enzyme–substrate-inhibitor)^23^.

In our previous studies, we first interrogated the existing tricyclic coumarin-based MTHFD2 inhibitors by extensive computational modeling that further guided us to perform structure-based drug design of new and selective analogues^24^. We furthermore analysed the conformational changes and selectivity elements associated with xanthine derivatives on binding to the allosteric site of MTHFD2^25^. In the present study, we have exclusively focused on the diaminopyrimidine-based inhibitors that bind to the substrate binding sites of MTHFD1, MTHFD2 and MTHFD2L. The available X-ray structures of MTHFD1, MTHFD2 and MTHFD2L in complex with diaminopyrimidine-based inhibitors **1–3** were thoroughly investigated by molecular dynamics simulations. The crystallographic binding modes, protein–ligand interactions and conformational dynamics were studied to establish a correlation with potent pharmacological activities. In addition, the binding modes of the diaminopyrimidine-based inhibitors were predicted by molecular docking and subsequent MD simulations for the compounds lacking published X-ray structures. We furthermore performed a comparative analysis on the binding mode of diaminopyrimidine-based inhibitor **1** with the existing tricyclic coumarin-based selective MTHFD2 inhibitor **5** which provided useful insights on the desirable and undesirable structural elements that must be taken into consideration to develop promising and selective inhibitors of MTHFD2.

## Materials and methods

### Protein preparation

The X-ray crystal structures of MTHFD1 in complex with LY345899 (PDB code: 6ECQ)^26^; MTHFD2 in complex with compound **1** (PDB code: 6S4A)^5^, compound **2** (PDB code: 6S4E)^5^ and compound **3** (PDB code: 6S4F)^5^; and MTHFD2L in complex with compound **2** (PDB code: 7QEI)^5^ were downloaded from the protein data bank^27^. The X-ray structures were prepared using the protein preparation wizard^28^ of Maestro, Schrödinger^29^. Missing bond orders were assigned based on geometry and using the chemical component directory (CCD). Optimization of hydrogen bonds was carried out which involves adding hydrogen atoms and addressing overlapping hydrogens in the aforementioned protein structures. Options to add zero order bonds between metals and nearby atoms, assigning correct formal charges to metal and neighboring atoms were kept on as a default setting, however, no metal ion was detected in the X-ray structures of the discussed proteins. In order to add missing atoms, sidechains and loops^28,30,31^ to the X-ray complexes, the Prime module^32^ of Schrödinger was employed. In cases where residues had alternate positions, the first listed position, or the position with the highest average occupancies, was selected. The PROPKA module^33^ of Schrödinger was used for assigning protonation states and generation of tautomeric states for Asp, Glu, Arg, Lys and His at pH 7.0 ± 2.0. As a part of optimizing H-bond networks, PROPKA alters ionization and tautomeric states of the protein residues, assigning relative penalties for different protonation states obtained from the pKa estimates. Water molecules with fewer than two hydrogen bonds to non-waters were deleted. Geometry refinements of all protein–ligand complexes were performed using the OPLS4 (Optimized Potential for Liquid Simulations) force field^34–37^ in restrained minimizations, in order to remove molecular overlaps and strains. The process of restrained minimization involves allowing hydrogen atoms to be freely minimized, facilitating sufficient heavy-atom movement to relax strained bonds, angles, and clashes. The restrained minimizations were terminated when the average root mean square deviation (RMSD) of the protein heavy atoms had converged to 0.3 Å.

### Ligand preparation

The diaminopyrimidine-based inhibitors **1–3** were prepared using the LigPrep module, as implemented in the Schrödinger suite. The pKa prediction tool Epik^38,39^ was employed to assign possible ionization and tautomeric states at pH 7.0 ± 2.0. Energy minimizations of the ligands were carried out employing the OPLS4 force field^34^.

### Molecular docking

Glide^40,41^ was used to perform the docking experiments, employing the ligand structures as prepared above. The prepared X-ray structures of MTHFD1–LY345899 (PDB code: 6ECQ), MTHFD2—compound **1** (PDB code: 6S4A), MTHFD2—compound **2** (PDB code: 6S4E), MTHFD2—compound **3** (PDB code: 6S4F) and MTHFD2L—compound **2** (PDB code: 7QEI) were used to generate receptor grids of their corresponding substrate binding sites. Cubic receptor grids were generated with respect to the centroid of the bound ligand in the MTHFD substrate binding sites, with a side length of 20 Å. The generated receptor grids allowed us to dock compounds **1–3** to the substrate binding sites of MTHFD1, MTHFD2 and MTHFD2L, under default settings without applying any receptor grid constraints. The standard precision (SP) mode of Glide docking was employed. This considers flexible sampling, nitrogen inversions and ring conformations of the ligands. The default parameters of a scaling factor of 0.8 for van der Waals radii of nonpolar ligand atoms and a 0.15 partial charge cutoff were used. Post-docking minimization was carried out, and a maximum of 10 poses per ligand were retained during the docking procedure. The top-ranked docking poses, scored by the default Glide scoring function, were finally selected for each ligand. The OPLS4 force field^34^ was used during all docking experiments. The reliability of the docking protocol was assessed by performing redocking of the co-crystallized inhibitors: LY345899 in MTHFD1, compounds **1–3** in MTHFD2, and compound **2** in MTHFD2L, that reproduced the crystallographic poses in each case, with only marginal RMSD differences.

### MD simulations and clustering

Starting from the co-crystallized pose/suggested docking pose of the diaminopyrimidine-based inhibitors **1–3** with respect to the MTHFD1, MTHFD2 and MTHF2L isoforms, classical MD simulations were carried out using the Desmond program^42^ as implemented in the Schrödinger suite. Na^+^ or Cl^−^ ions were added as appropriate to adjust the electroneutrality of the system. A salt concentration of 0.15 M NaCl was added to the simulation box to reproduce the physiological conditions. The default NPT ensemble from the Desmond package was used for minimization and relaxation of each system within the default settings. The default relaxation protocol includes four stages of minimization (restrained and unrestrained) as following:

1. Brownian dynamics NVT, T = 10 K, restraints on solute heavy atoms, 100 ps.
2. NVT, T = 10 K, restraints on solute heavy atoms, 12 ps.
3. NPT, restraints on solute heavy atoms, 12 ps.
4. NPT, no restraints, 24 ps.

The OPLS4 (Optimized Potential for Liquid Simulations) force field^34^ that has been developed to evaluate small-molecule solvation and protein–ligand binding, was applied during all simulations. A buffer distance of 10 Å was used to solvate the structures with TIP3P water using an orthorhombic box with periodic boundary conditions. For all the systems, each simulation was run for 200 ns with a recording interval of 200 ps. The temperature and pressure of the system were maintained constant at 300 K and 1.01325 bar atmospheric pressure, employing the Nose–Hoover thermostat and Martyna–Tobias–Klein barostat with isotropic coupling^43–45^, respectively. Root mean square deviations (RMSD) and protein–ligand interactions were analysed using the simulation interaction diagram (SID) panel as implemented in Maestro, Schrödinger. All simulations were performed in triplicates. The replica showing the best stability with respect to each protein—inhibitor complex was selected as the representative replica and further analysed. As observed from the triplicate MD simulation of MTHFD2—inhibitor complexes, it was found that either all replicas show a great similarity with each other or one of the three replicas was dissociative, whereas the other two behaved similarly, eventually verifying the reliability of our MD simulation protocol. The “Desmond Trajectory Clustering” module was used to cluster the MD trajectories based on RMSD while setting up a frequency value of 10 (every 10th ns) and generating up to a maximum of 10 clusters. The most populated cluster obtained from the trajectory clustering of each MTHFD—inhibitor complex was used as the representative structure.

### Binding free energy calculation

We have employed a widely used method ‘Molecular mechanics with generalized Born and surface area solvation’ (MM-GBSA); to compute the binding free energy of a ligand, bound to a protein. The Prime module^32^ in the Schrödinger suite 2023-2^29^ was employed to compute MM-GBSA free energy of binding (ΔG bind) for the crystallographic and docking poses, with respect to MTHFD1, MTHD2 and MTHFD2L isoforms, using the following equation:

$$\Delta G \, bind \, = \, E_{Complex} {-} \, E_{Ligand} {-} \, E_{Receptor} ,$$

where E_Complex_, E_Ligand_, and E_Receptor_ represent the energies calculated in the Prime MM GBSA module for the optimized complex (complex), optimized free ligand (ligand), and optimized free receptor (receptor), respectively. The OPLS4 force field^34–36^ and VSGB solvation model were used in the calculations, featuring minimization of protein–ligand complexes as the sampling method. The binding free energies (ΔG Bind), H-bond energy contribution (ΔG Hbond), Lipophilic energy (ΔG Lipophilic) and van der Waals energy (ΔG van der Waals) of each ligand are discussed in the present study.

## Results and discussion

### Availability of X-ray crystal structures

All three diaminopyrimidine-based inhibitors were co-crystallized with MTHFD2: compound **1** (PDB code: 6S4A), compound **2** (PDB code: 6S4E) and compound **3** (PDB code: 6S4F). With regards to MTHFD2L, compound **2** is the only co-crystallized inhibitor (PDB code: 7QEI)^5^. The X-ray structure of MTHFD1 in complex with LY345899 and NADP (PDB code: 6ECQ)^26^ was also used in this study for docking and subsequent post-docking analysis. The X-ray structure of MTHFD2 in complex with a tricyclic coumarin-based selective inhibitor (PDB code: 6KG2, compound **5**)^22^ was employed in the present work to compare with the crystallographic binding mode of compound **1**.

### Comparison of MTHFD1, MTHFD2 and MTHFD2L substrate binding sites

**Fig. 2:**
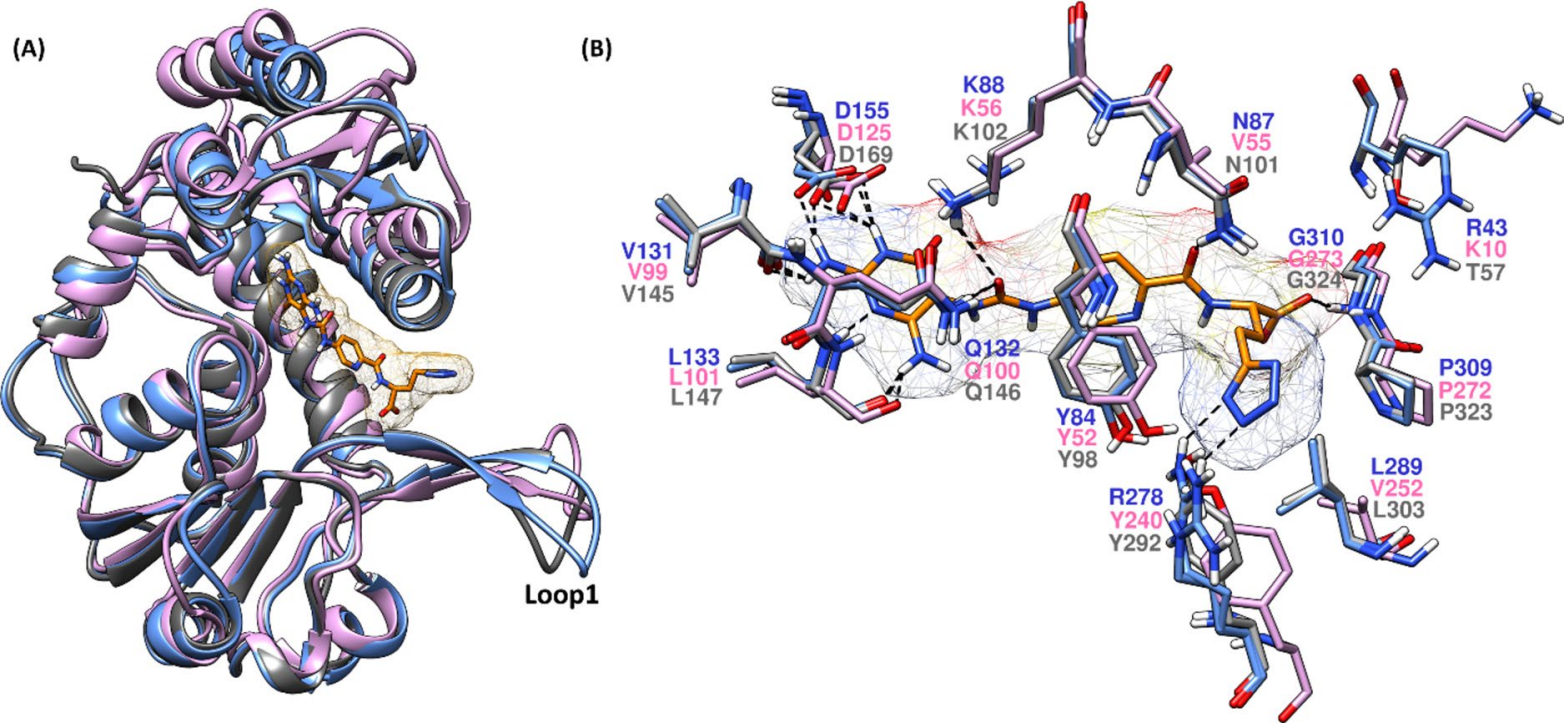
(**A**) Superposed X-ray structures of MTHFD1 (PDB code: 6ECQ, monomer A, pink ribbons), MTHFD2 (PDB code: 6S4A, monomer A, blue ribbons) and MTHFD2L (PDB code: 7QEI, monomer A, grey ribbons). Diaminopyrimidine-based inhibitor **1** (orange) is co-crystallized in the substrate binding site in 6S4A. (**B**) Substrate binding site residues superposed and labelled (MTHFD1—pink, MTHFD2—blue, MTHFD2L—grey), with compound **1** shown in orange.

As discussed earlier, MTHFD2 is exclusively expressed in several cancer types such as breast cancer^2^, colorectal cancer^3^, acute myeloid leukaemia^4,5^, renal cell carcinoma^6^ and hepatocellular carcinoma^7^, while absent in healthy adult human tissues, which makes MTHFD2 an important target for cancer drug discovery. On the other hand, the two close homologs: MTHFD1 and MTHFD2 are present in healthy adult human tissues, thus rendering the development of selective MTHFD2 inhibitors more challenging. To understand the non-selective binding modes of the reported diaminopyrimidine-based inhibitors^5^, the X-ray structures of MTHFD1, MTHFD2 and MTHFD2L were superposed and compared with each other at the substrate binding site level. In Fig. 2, the X-ray structure of MTHFD2 in complex with compound **1** (PDB code: 6S4A) is superposed with the X-ray structure of MTHFD1 bound to LY345899 and NADP (PDB code: 6ECQ), and the X-ray structure of MTHFD2L bound to compound **2** (PDB code: 6S4E). The central pyridine ring and the diaminopyrimidine scaffold of **1** accommodates well into the substrate binding site of all three while its terminal tetrazole unit is projected towards the solvent-exposed region, in the vicinity of loop 1. As reported in our previous work^24^, Arg43 in MTHFD2 is a key residue that can be exploited for selectivity because the corresponding MTHFD1 and MTHFD2 residues (Lys10 and Thr57, respectively) are projected outward from the substrate binding site and are inaccessible for H-bond contact with the inhibitor. The butanoic acid unit of the pyrimidine-based compound **1** forms H-bond interactions with the backbone nitrogens of Gly273 (MTHFD1), Gly310 (MTHFD2) and Gly324 (MTHFD2L). The two nitrogens from the tetrazole moiety of **1** establish salt-bridge contacts with the sidechain of Arg278 in MTHFD2, however, no H-bond/salt-bridge interaction is observed with MTHFD1 and MTHFD2L at the same position due to the fact that the longer and flexible Arg278 is replaced by structurally different residues Tyr240 in MTHFD1 and Tyr92 in MTHFD2L, providing promising further scope of selective MTHFD2 binding. The pyridine ring of **1** demonstrates π–π stacking with Tyr52 (MTHFD1), Tyr84 (MTHFD2) and Tyr98 (MTHFD2L), contributing to the high binding affinities for the three isoforms. Interestingly, the hydrophilic asparagine in MTHFD2 (Asn87) and MTHFD2L (Asn101) are replaced by lipophilic valine (Val55) in MTHFD1. The amide carbonyl of **1** H-bonds to both Asn87 and Asn101 whereas no interaction was noted between the inhibitor and Val55 in MTHFD1 which renders selectivity potential. The urea carbonyl of compound **1** establishes H-bond interaction with Lys56 and Gln100 in MTHFD1, Lys88 and Gln312 in MTHFD2, and Lys102 and Gln146 in MTHFD2L, respectively. Finally, the diaminopyrimidine core of **1** seems to be an important structural element and is projected inward of the substrate binding site, forming identical H-bonds to all three MTHFD isoforms. One of the amino groups of the compound **1** diaminopyrimidine scaffold H-bonds to the backbone oxygen of Leu101, Leu133 and Leu147 of MTHFD1, MTHFD2 and MTHFD2L respectively, while its other amino group engages in H-bond contacts with Val99 and Asp125 (MTHFD1), Val131 and Asp155 (MTHFD2), and Val145 and Asp169.

Despite that the diaminopyrimidine scaffold seems to contribute crucially to the binding affinity for the MTHFD isoforms, it is not advisable to use in selective MTHFD2 inhibitor design due to its interior placement in the substrate binding site where all MTHFD residues are identical and do not provide any scope for selectivity. Taken together, avoiding scaffolds like diaminopyrimidine in the rational structure-based design of new MTHFD2 inhibitors, instead targeting Arg43, Asn87 and Arg278 appears to open up new opportunities to attain selectivity (Table 1).

**Table 1:** Residue comparison at the substrate binding site of MTHFD1, MTHFD2 and MTHFD2L.

Entry	MTHFD1	MTHFD2	MTHFD2L
1.	Lys10	Arg43*	Thr57
2.	Tyr52	Tyr84	Tyr98
3.	Val55	Asn87**	Asn101
4.	Lys56	Lys88	Lys102
5.	Val99	Val131	Val145
6.	Gln100	Gln132	Gln146
7.	Leu101	Leu133	Leu147
8.	Asp125	Asp155	Asp169
9.	Gly273	Gly310	Gln324
10.	Tyr240	Arg278*	Tyr292

*MTHFD2 residues different to MTHFD1 and MTHFD2L, important for selectivity.

**MTHFD2 residue different to MTHFD1, however, similar to MTHFD2L, important for selectivity.

### Docking and MD simulations of compounds 1–3 bound to MTHFD1

Due to the unavailability of X-ray structures of MTHFD1 in complex with diaminopyrimidine analogues, we first performed molecular docking of compounds **1–3** in the substrate binding site of MTHFD1. As illustrated in Table 2, all compounds show high binding affinity with MTHFD1, possessing Glide docking scores of better than − 10.0 kcal/mol, further correlating with the experimental results. Similar to the aforementioned discussion on the superposed MTHFD structures (with the co-crystallized pose of compound **1** in MTHFD2), the suggested docking pose of **1** shows the desirable binding mode and existence of multiple protein–ligand contacts in the substrate binding site of MTHFD1 (Figs. S2A, S3). The butanoic acid unit of inhibitor **1** H-bonds to the backbone nitrogen of Gly273 while the central pyridine ring forms π–π stacking with Tyr52. Lys56 of MTHFD1 is involved in H-bond interactions with the urea carbonyl and diaminopyrimidinone carbonyl of **1**. As expected, the diaminopyrimidine scaffold of compound 1 is placed inward to the substrate binding site of MTHFD1, constituting multiple H-bond contacts with Val99, Leu101, Asp125. Only the tetrazole unit of **1** is oriented away from the binding site, towards Lys10 but not forming any interactions. Compound **2**, which is characterized by the presence of a central phenyl ring instead of the pyridine ring in **1**, displays a similar binding mode and identical protein–ligand interactions in the MTHFD1 binding site (Figs. S2B, S4). In comparison with compound **1**, the central 3-fluororpyridine ring in compound **3** is placed slightly away from Tyr52, resulting in loss of lipophilic contact; however, this is compensated by the presence of two additional H-bond interactions; one between glutamic acid sidechain of **3** and Gly274, and another between the amide carbonyl of **3** and Gln100, which overall contribute to the improved docking score of − 10.75 kcal/mol (Figs. S2C, S5).

**Table 2:** Glide scores (in kcal/mol) of **1–3** in MTHFD1, MTHFD2 and MTHFD2L.

Entry	MTHFD1	MTHFD2	MTHFD2L
Compound 1	− 10.51	− 12.66	− 11.90
Compound 2	− 10.65	− 12.65	− 11.43
Compound 3	− 10.75	− 12.50	− 12.22

**Fig. 3:**
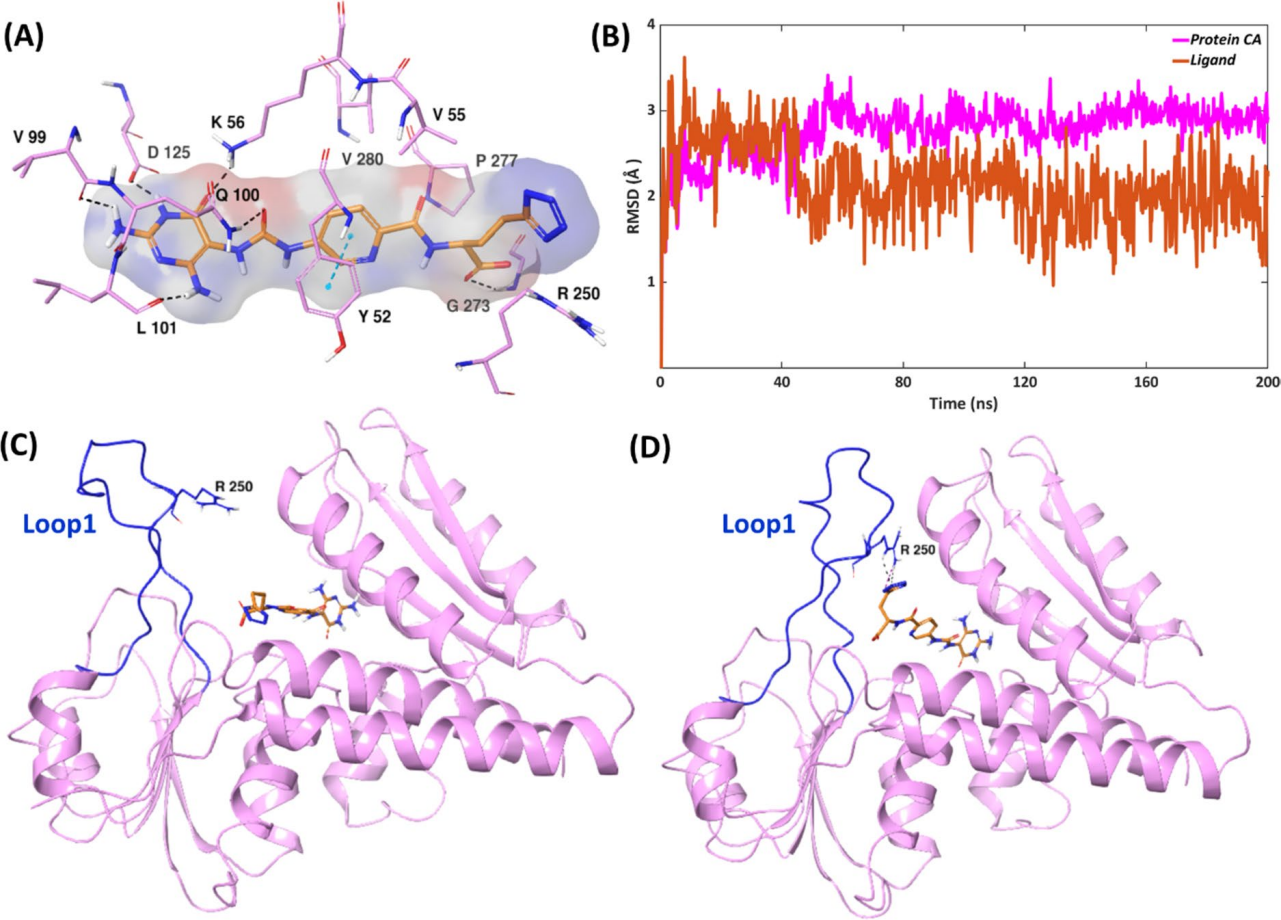
(**A**) Representative MD structure of the MTHFD1—compound **1** complex (inhibitor in orange, protein residues in pink). (**B**) RMSD analysis of the MTHFD1—compound **1** complex (ligand in orange, protein α-carbons in pink) during the 200 ns simulation. Ribbon view of the MD structures of MTHFD1—compound **1** complex: (**C**) at 0 ns, (**D**) at 15 ns. MTHFD1 ribbons in pink, loop 1 in dark blue and **1** in orange.

In order to evaluate the conformational dynamics, binding mode, and stability of protein–ligand interactions, 200 ns molecular dynamics (MD) simulations were performed of MTHFD1 in complex with each inhibitor starting from their suggested docking poses. As a measure of protein mobility, the Root Mean Square Deviation (RMSD) of the α-carbons of the protein was calculated throughout the simulation. RMSD of the ligand heavy atoms during the course of the simulation was also calculated as a measure of ligand mobility. The RMSD depicts the deviation of the ligand/protein α-carbon atoms from their initial suggested docking pose during the simulations. All MD simulations with respect to the 3 isoforms (MTHFD1, MTHFD2 and MTHFD2L) were performed in triplicates with the purpose of re-evaluating the stability of the MTHFD—inhibitor complexes. The triplicate RMSD plots of the MTHFD1—compound **1** complex indicate significant stability over the course of the simulation, showing marginal differences among all replicas, with respect to both MTHFD1 α-carbons and compound **1** movement (Fig. S6). The overall average RMSDs calculated from the triplicate MD simulations of the MTHFD1—compound **1** complex were found to be 2.2 Å and 2.5 Å for the MTHFD1 α-carbons and compound **1**, respectively (Table S1). Replica 2 was selected as the representative replica for further individual analysis. In this replica, compound **1** demonstrated a decent stability in the substrate binding site of MTHFD1, with an average RMSD of 2.2 Å, while the MTHFD1 α-carbons possess mildly higher average RMSD of 2.8 Å (Fig. 3A,B). MD analysis furthermore verified the stability of the docking pose of compound 1. The initial minor fluctuations between 0 and 50 ns of the MD trajectory correspond to the dynamic movement of the butyl-tetrazole unit of **1**. The interactions with Lys56, Leu101 and Asp 125 of MTHFD1 were maintained for 100% of the simulation time, while the interactions with Tyr52, Val99, Gln100 and Gly274 account for 94%, 68%, 67% and 70% of the simulation time, respectively. In addition to the aforementioned protein–ligand interactions observed from the docking pose, the MD analysis also revealed lipophilic contact between the central pyridine ring of the inhibitor and Val280 for 50%, and H-bond/salt-bridge interaction between the compound **1** tetrazole unit and Arg250 for 36% of the simulation time (Fig. S7).

Interestingly, loop 1 of MTHFD1 displayed some conformational changes when inhibitor **1** was bound to the substrate binding site (Fig. 3C,D, Fig. S8). The initial docking pose and the energy minimized structure suggest that loop 1 is oriented away from **1** without establishing any interactions. However, the loop was found to fluctuate towards **1** already from the first 15 ns onwards, establishing H-bond and salt-bridge contacts between Arg250 and the tetrazole unit of the inhibitor. We also superposed the MD structure of the MTHFD1—compound **1** complex with the X-ray structure of MTHFD1 without inhibitor (PDB code: 6ECR), with the purpose of verifying the observed conformational changes. As shown in Fig. S9, in the 6ECR MTHFD1 structure, loop 1 seems to be notably away from the position it attains in the MTHFD1-compound **1** complex, which verifies the crucial conformational changes taking place. We furthermore hypothesize that the butyl-tetrazole unit of **1** is an important element in facilitating the conformational changes in loop 1 by interacting with Arg250, overall correlating with the 0.5 nM IC_50_ of inhibitor **1** against MTHFD1.

**Fig. 4:**
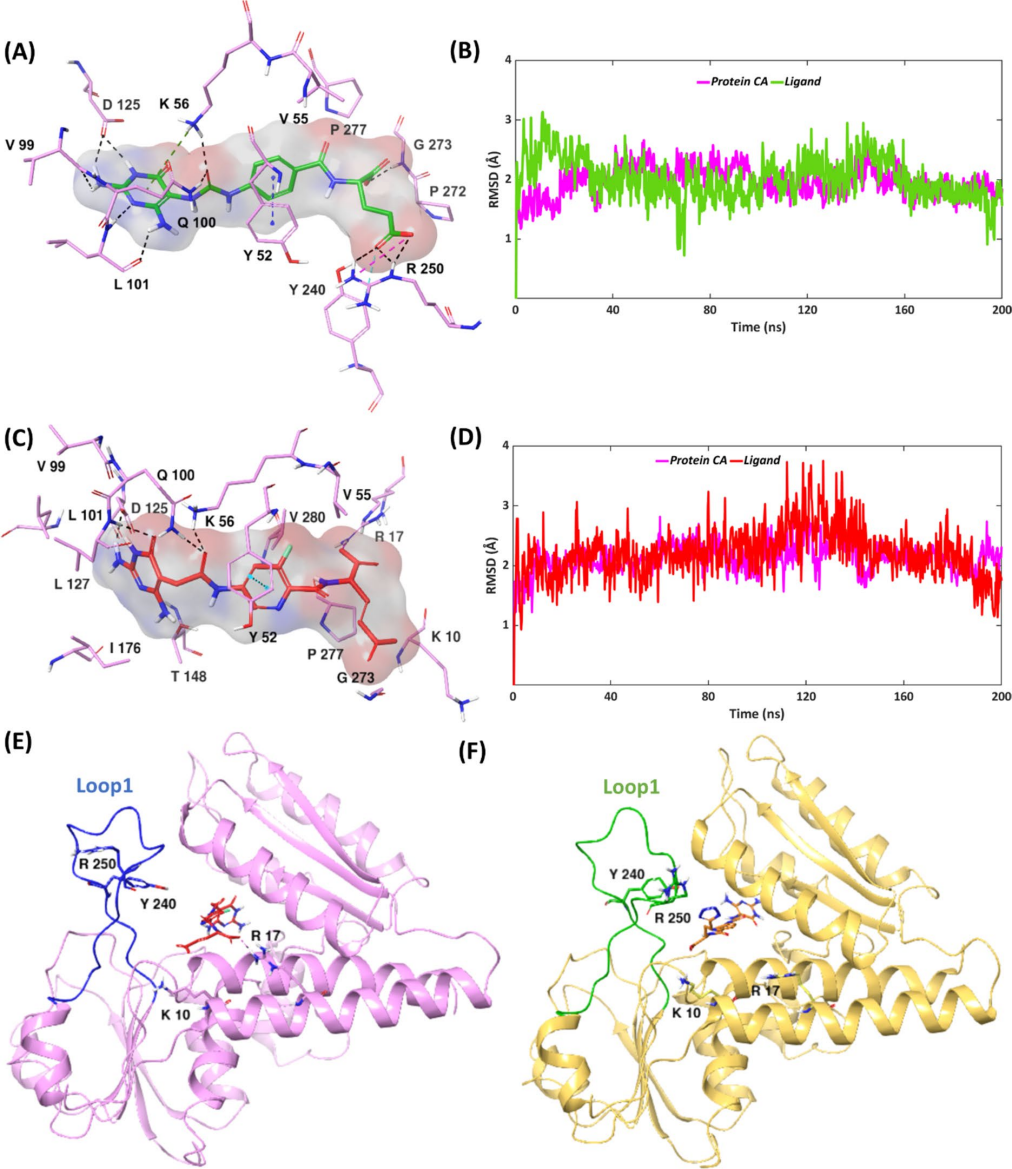
Representative MD structures of (**A**) MTHFD1—compound **2** complex (inhibitor in green, protein residues in pink). (**C**) MTHFD1—compound **3** complex (inhibitor in red, protein residues in pink). RMSD analysis of (**B**) MTHFD1—compound **2** complex (ligand in green, protein α-carbons in pink). (**D**) MTHFD1—compound **3** complex (ligand in red, protein α-carbons in pink) during the 200 ns MD simulation. (**E**) Representative MD structure of the MTHFD1—compound **3** complex. MTHFD1 protein ribbons are shown in pink, loop 1 in dark blue and **3** in red. Compound **3** is in close proximity to Lys10 and Arg17 of the substrate binding site, but far from Tyr240 and Arg250 of loop 1. (**F**) Representative MD structure of the MTHFD1—compound **1** complex. MTHFD1 protein ribbons are shown in golden, loop 1 in green and **1** in orange. Compound **1** is in close proximity to Tyr240 and Arg250 of loop 1, but far from Lys10 and Arg17 of the substrate binding site.

As observed from the triplicate MD simulations of MTHFD1—compound **2**, the MTHFD1 α-carbons show decent stability and similar pattern of convergence, with an overall average RMSD of 2.2 Å. Furthermore, an overall average RMSD of 3.2 Å was noted for ligand replicas. Replicas 1 and 2 of compound **2** superpose almost perfectly during the simulation, however, replica 3 demonstrates drastic fluctuation (Fig. S10, Table S2). Replica 1 was selected as representative for further discussion. The MTHFD1—compound **2** complex possesses notable stability over the course of the simulation, with mild initial fluctuations and 1.9 Å and 2.0 Å average RMSDs for ligand and protein, respectively (Fig. 4A,B). Similar to the MTHFD1—compound 1 complex, engagement with the MTHFD1 loop1 is noted when the diaminopyrimidine-based compound 2 is bound, establishing H-bond/salt-bridge interactions with Tyr240 and Arg250, that were maintained for 39% and 61% of the simulation time, respectively (Figs. S11, S12). However, the dynamic movement of the MTHFD1 loop1 in presence of **2** is limited compared to what was observed for compound **1**. It can thus be hypothesized that the conformational changes at loop 1, particularly the movement towards the substrate binding site, are more favored in the proximity of tetrazole (compound **1**) whereas in presence of glutamic acid (compound **2**), the inward projection of loop 1 is relatively restricted, which is in good agreement with 89 nM IC_50_ of **2** against MTHFD1 (as compared to 0.5 nM IC_50_ of **1**). Interactions of **2** with Lys56, Leu101 and Asp125 of MTHFD1 exist during the whole simulation while the interactions with Tyr52, Val99, Gln100 and Gly273 account for 89%, 70%, 84% and 86% of the simulation time, respectively.

The triplicate MD simulations of the MTHFD1—compound **3** complex show an overall average RMSD of 2.6 Å and 3.4 Å for MTHFD1 α-carbons and compound **3**, respectively, demonstrating significant stability (Fig. S13, Table S3). Only replica 2 of compound **3** exhibits some fluctuations between ∼ 150 and 180 ns, but otherwise algins well with other replicas. Replica 3 was chosen as representative from these simulations. The MTHFD1—compound **3** complex possesses significant stability during the 200 ns simulation, with both the MTHFD1 α-carbons and compound **3** showing a high convergence, maintaining an average RMSD of 2.1 Å and 2.2 Å, respectively (Fig. 4C,D). Unlike **1** and **2**, compound **3** does not interact with loop 1. Instead, it is located somewhat deeper into the substrate binding site of MTHFD1, forming additional H-bond interactions that were not observed with compounds **1** or **2**; the backbone oxygen of the glutamic acid of **3** forms H-bond/salt-bridge contact with Arg17 for 90% of the simulation time while its carboxy sidechain forms an H-bond with Lys10 during 37% of the simulation (Fig. 4E, Fig. S14). On comparing the MD pose of the MTHFD1—compound **3** complex with the MTHFD1—compound **1** complex (Fig. 4E,F), we note that the acyclic glutamic acid unit of **3** facilitates interaction with Lys10 and Arg17 instead of interacting with Tyr240 and Arg250, thereby limiting the interaction with loop 1. The flexible butyl-tetrazole unit of **1**, on the other hand, is projected away from the Lys10 and Arg17 substrate site residues and engages with Arg250, facilitating the notable movement and conformational changes in loop 1. The distinct interactions of **3** with Lys10 and Arg17 thus compensate for the absence of interactions with loop1 and are hypothesized to crucially contribute to its high binding affinity (IC_50_ 16 nM) in addition to the essential interactions with Tyr52 and Lys56 (100% of the simulation time), and interactions with Gln100, Leu101, Asp125, Gly273 that exist for 96%, 80%, 97% and 37% of the simulation, respectively. The central 3-fluoropyridine ring system establishes lipophilic contacts with Val55 and Val280 for 32% and 44% of the simulation trajectory, respectively. The presence of the 3-fluoro atom also facilitates interactions with Val55 and Val280 of MTHFD1, overall contributing to the improved binding affinity.

### MD simulations of compounds 1–3 bound to MTHFD2

**Fig. 5:**
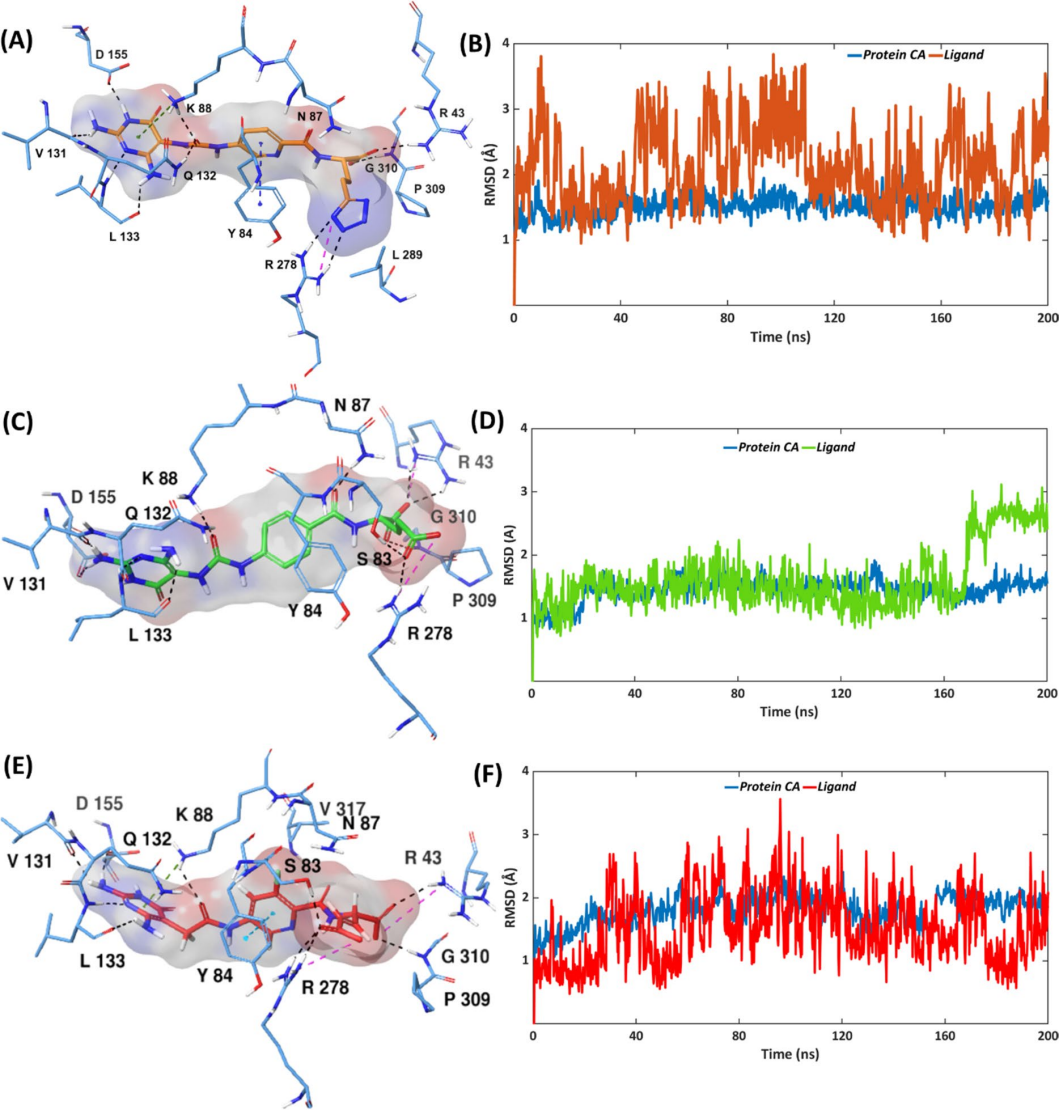
Representative MD structures of (**A**) MTHFD2—compound **1** complex (inhibitor in orange, protein residues in blue). (**C**) MTHFD2—compound **2** complex (inhibitor in green, protein residues in blue). (**E**) MTHFD2—compound **3** complex (inhibitor in red, protein residues in blue). RMSD analysis of (**B**) MTHFD2—compound **1** complex (ligand in orange, protein α-carbons in blue). (**D**) MTHFD2—compound **2** complex (ligand in green, protein α-carbons in blue). (**F**) MTHFD2—compound **3** complex (ligand in red, protein α-carbons in blue) during the 200 ns simulation.

The co-crystallized binding poses of compounds **1–3** in MTHFD2 (Figs. S16–S19, PDB codes: 6S4A, 6S4E and 6S4F for compound **1, 2** and **3**, respectively) were subjected to triplicate 200 ns MD simulations. Both the protein α-carbons and compound **1** possess remarkable stability during the triplicate MD simulations, demonstrating overall average RMSDs of 1.7 Å and 2.0 Å, respectively, (Fig. S15, Table S4). Replica 1 was selected as representative. The MTHFD2—compound **1** complex exhibits significant stability over the course of the simulation, with the MTHFD2 α-carbons and the diaminopyrimidine-based compound **1** possessing average RMSDs of 1.5 Å and 2.1 Å, respectively (Fig. 5A,B). Compound **1** illustrates some fluctuations throughout the simulation which correspond to its flexible butyl-tetrazole unit.

**Fig. 6:**
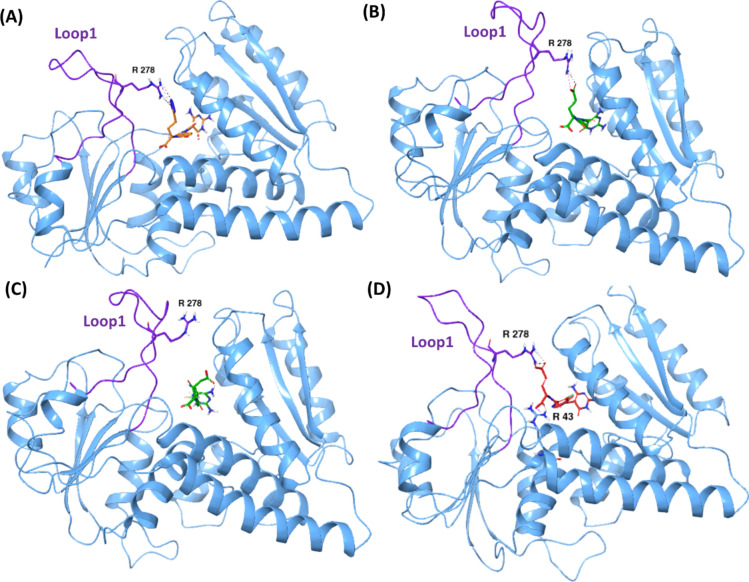
(**A**) Representative MD structure of the MTHFD2—compound **1** complex, showing H-bond with Arg278. (**B**) MD snapshot of the MTHFD2—compound **2** complex at 136 ns, showing H-bond with Arg278. (**C**) MD snapshot of the MTHFD2—compound **2** complex at 175 ns, showing no H-bond with Arg278. (**D**) Representative MD structure of the MTHFD2—compound **3** complex, showing H-bond with Arg43 and Arg278. MTHFD2 ribbons are shown in blue, loop 1 in purple, compound **1** in orange, **2** in green and **3** in red.

As observed from the simulation trajectory, loop 1 of MTHFD2 shows limited movement in presence of **1** (as compared to loop1 of the MTHFD1—compound **1** complex). Tyr240 in the MTHFD1 loop1 is replaced by the longer, bulkier and flexible Arg278 in loop 1 of MTHFD2, which is more accessible to the diaminopyrimidine-based inhibitors (Fig. 2B). The tetrazole unit of **1** H-bonds with Arg278 for 63% of the simulation, indicating a significant engagement to loop 1, however, with less movement (Figs. 5A, 6A, Fig. S20). Furthermore, the H-bond contacts between the amide carbonyl of **1** and Asn87, and between the diaminopyrimidine ring of **1** and Leu133 and Asp155, were maintained for the whole simulation time. Lys88 of MTHFD2 H-bonds to the urea carbonyl of compound **1** for 90%, and further engages its diaminopyrimidine scaffold in a π–cation interaction for 92% of the simulation. In addition, the urea carbonyl of **1** is engaged in H-bond interaction with Gln132 for 80% of the simulation trajectory. The diaminopyrimidine ring of **1** H-bonds to Val131 for 88% of the simulation time and the backbone oxygen of the compound **1** glutamic acid H-bonds to Gly310 for 81% of the simulation time. Finally, lipophilic contacts of the central pyridine ring of **1** with Tyr84 and Val317 exist for 85% and 58% of the simulation. The overall strong interaction profile along with engagement to Arg278 of loop 1 contribute to the high binding affinity for MTHFD2 (IC_50_ 16 nM).

As shown in the triplicate MD simulations of the MTHFD2—compound **2** complex (Fig. S21, Table S5), the overall average RMSD of MTHFD2 α-carbons was found to be 2.0 Å, facilitating remarkable stability and similar pattern of convergence. Replicas 1 and 2 of compound **2** aligns perfectly, while replica 3 completely dissociates from the substrate binding site of MTHFD2. Replica 2 was chosen as the representative replica for further analysis. Compound **2** also displays a great stability at the substrate binding site of MTHFD2 during the simulation, possessing an average RMSD of 1.6 Å; albeit with a sudden jump in RMSD between 167 and 172 ns. The MTHFD2 α-carbons were found to be consistently stable over the course of the simulation, possessing an average RMSD of 1.4 Å (Fig. 5C,D). The aforementioned RMSD fluctuations in the interval between 167 and 172 ns correspond to a couple of conformational changes: (a) movement of the glutamic acid sidechain of **2**, contributing to H-bond interaction with Asn87, and (b) rotation of the central phenyl ring of **2**, thereby forming π–cation interaction with Lys88. In addition, apart from the glutamic acid sidechain, the amide carbonyl of **2** H-bonds to Asn87 resulting in an overall H-bond interaction with Asn87 for 100% of the simulation. Likewise, the urea carbonyl of compound **2** H-bonds to Lys88, including the above-mentioned π–cation interaction, leads to a resulting interaction between these for 85% of the simulation time. The H-bond interactions between the diaminopyrimidine ring of **2** and Leu133 and Asp155 exist for 100% of the simulation. H-bond interaction of the diaminopyrimidine system of compound **2** with Val131 furthermore accounts for 80% of the simulation while π–π stacking between the central phenyl ring of **2** and Tyr84 was maintained for 73% of the simulation. Finally, the H-bond contact between the urea carbonyl and Gln132 is maintained for 90% of the simulation trajectory (Fig. S22).

Despite a desirable interaction profile at the substrate binding site of MTHFD2, compound **2** exhibited negligible contact with loop1 of MTHFD2, possessing H-bond interaction with Arg278 just for 17% of the simulation time (Fig. 6B,C). The limited interaction of compound **2** with loop1 of MTHFD2 can be thus correlated with the lower IC_50_ of 254 nM as observed from the biochemical screening.

Average RMSDs of 1.6 Å and 2.7 Å were observed from the triplicate MD simulations of MTHFD2—compound **3** for protein α-carbons and ligand, respectively (Fig. S23, Table S6). The MTHFD2 α-carbons in all replicas demonstrated great stability and identical convergence, whereas replica 1 and replica 2 of compound **3** show sudden jump at ~ 30 ns and ~ 100 ns, respectively. Replica 3 was selected as representative from the triplicate MD simulations of MTHFD2—compound **3**. Compound **3** displayed remarkable stability at the MTHFD2 substrate binding site over the course of the simulation, with an average RMSD of 1.5 Å. The protein α-carbons also showed very low average RMSD of 1.8 Å (Fig. 5E,F). The fluctuations of **3**, as observed from the RMSD plots, correspond to the dynamic movement of its glutamic acid sidechain. The H-bond interactions between the diaminopyrimidine scaffold of **3** and Val131, Leu133 and Asp155 account for 91%, 100% and 100% of the simulation time, respectively (Fig. S24), and π–π stacking between the central 3-fluoropyridine ring of **3** and Tyr84 exists during 80% of the simulation. Lys88 engages the amide carbonyl of compound **3** (near to the diaminopyrimidine system) via H-bond interaction for 80%, and with the diaminopyrimidine ring via π–cation interaction for 60% of the simulation trajectory, thus significantly contributing to the binding affinity towards MTHFD2. Moreover, the glutamic acid sidechain of **3** engages with loop1 of MTHFD2, establishing H-bond interaction with Arg278 for 43% of the simulation time (Fig. 6D). Unlike compounds **1** and **2**, the H-bond contact between the amide carbonyl (near the glutamic acid) of compound **3** and Asn87 was reduced to only 35% of the simulation time. The interaction profile of **3** with MTHFD2 including the reduced interaction with Asn87 and moderate engagement with loop 1 thus results in its 47 nM inhibitory activity against MTHFD2.

### Docking and MD simulations of compounds 1–3 bound to MTHFD2L

**Fig. 7:**
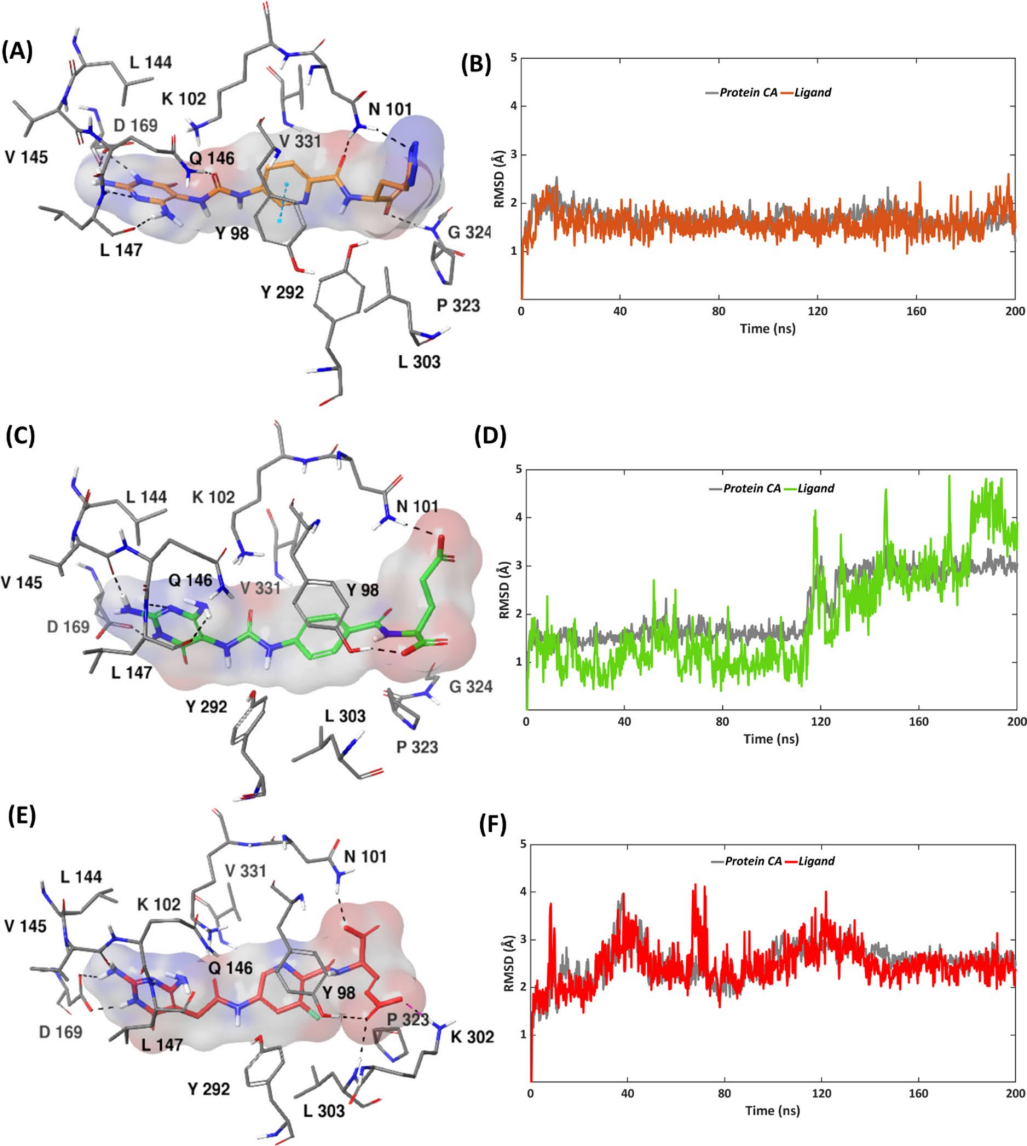
Representative MD structures of (**A**) MTHFD2L—compound **1** complex (inhibitor in orange, protein residues in grey). (**C**) MTHFD2L—compound **2** complex (inhibitor in green, protein residues in grey). (**E**) MTHFD2L—compound **3** complex (inhibitor in red, protein residues in grey). RMSD analysis of (**B**) MTHFD2L—compound **1** complex (ligand in orange, protein α-carbons in grey). (**D**) MTHFD2L—compound **2** complex (ligand in green, protein α-carbons in grey). (**F**) MTHFD2L—compound **3** complex (ligand in red, protein α-carbons in grey) during the 200 ns simulation.

The X-ray structure of MTHFD2L in complex with compound **2** is available in the protein data bank (PDB code: 7QEI) whereas for **1** and **3**, we performed molecular docking to obtain their putative binding poses (Figs. S25–S28). The suggested docking pose of inhibitor **1** shows the existence of plenty of H-bond contacts in the MTHFD2L binding site, similar to the binding sites of the other two isoforms. The triplicate MD simulations of the MTHFD2L—compound **1** complex demonstrated remarkable stability, showing overall average RMSDs of 1.9 Å and 1.7 Å for protein α-carbons and **1**, respectively, further selecting replica 3 as being representative (Fig. S29, Table S7). Both the protein α-carbons and compound **1** were highly stable during the simulation, possessing average RMSD’s of 1.7 Å and 1.6 Å, respectively (Fig. 7A,B). The terminal tetrazole unit of **1** H-bonds to Tyr292 of MTHFD2L loop 1; however, the said interaction was maintained for just 24% of the simulation time (Fig. S30). Interestingly, Asn101 engages both the amide carbonyl and the tetrazole unit of **1** via H-bond interactions which altogether lead to the existence of H-bond between the two during 100% of the simulation. π–π stacking between the pyridine ring of **1** and Tyr98 exists for 88% of the simulation. Lys102 H-bonds to the urea carbonyl of compound **1** for 90%, and further establishes π–cation interaction with its diaminopyrimidine ring for 71% of the simulation trajectory. The urea carbonyl of **1** furthermore H-bonds to Gln146 during 52% of the simulation time. H-bond contacts exist between the diaminopyrimidine scaffold of **1**, and Leu147 and Asp169, throughout the simulation, while interaction with Val145 is maintained for 89% of the simulation time. Despite the limited contact with the loop 1 of MTHFD2L and due to a sequence identity of 72% between MTHFD2 and MTHFD2L, the aforementioned interactions indicate a significant contribution to the improved binding affinity, correlating with the IC_50_ value of 27 nM for compound **1** against MTHFD2L as obtained in the biochemical screening.

Replica 3 was selected as the representative replica from the triplicate MD simulations of MTHFD2L—compound **2** complex. All replicas of MTHFD2 α-carbons demonstrated similar RMSD pattern with minor fluctuations, possessing an overall average RMSD of 2.2 Å. Only replica 3 of compound 2 seems to be reasonably stable, while replica 2 fluctuates significantly and replica 1 dissociates completely, leading to an overall average RMSD of 5.0 Å (Fig. S31, Table S8). In replica 3, compound **2** demonstrated significant stability for the first 114 ns of the simulation, followed by some notable fluctuations, giving an average RMSD of 1.9 Å. Correspondingly, a fluctuation between 113 and 125 ns was noted among the protein α-carbons, resulting in an average RMSD of 2.1 Å (Fig. 7C,D). The ligand fluctuations mainly corresponds to the dynamic movement of the flexible glutamic acid unit of **2** and to some extent the central phenyl ring, whereas the RMSD fluctuations of the MTHFD2L α-carbons represents dynamic movements of loop 1 and α-helix 1 (Fig. S32). The observed ligand instability and the limited loop 1 interaction are in correlation with the weaker MTHFD2L inhibitory activity of **2** against MTHFD2L with an IC_50_ of 126 nM. Compound **2** interacts with Asn101 for the whole duration of the simulation via H-bonds to its amide carbonyl and its glutamic acid moiety (Fig. S33). The aforementioned conformational changes allow Lys102 of MTHFD2L to interact with the urea carbonyl of **2** via H-bond formation (88%) and with the diaminopyrimidine scaffold via π-cation interaction (41%). In addition, the urea carbonyl of **2** forms H-bond interaction with Gln146 of MTHFD2L during 42% of the simulation. Tyr98 interacts with the central phenyl ring of **2** by π–π stacking for 68%, while it additionally H-bonds to the glutamic acid sidechain of **2** for 20% of the simulation time. H-bond interactions between the diaminopyrimidine scaffold of compound **2** and Val145, Leu147 and Asp169 account for 90%, 100% and 100% of the simulation trajectory, respectively. The H-bond contact between **2** and loop 1 of MTHFD2L is limited to only 31% of the simulation time, and is seen between the glutamic acid and Tyr292, whereas interaction between the backbone oxygen of compound **2** and Gly324 was observed for 55% of the simulation time.

Overall average RMSDs of 2.3 Å and 3.2 Å for protein α-carbons and inhibitor, respectively, were observed from the triplicate MD simulations of the MTHFD2L—compound **3** complex, indicating a stable protein and ligand movement (Fig. S34, Table S9). The protein replicas exhibit minor fluctuations, albeit some notable jumps were seen for the ligand in replicas 2 and 3. Replica 1 was selected as representative replica in this study. The MTHFD2L—compound **3** complex displayed significant stability during the course of the simulation, possessing average RMSDs of 2.5 Å and 2.4 Å for protein α-carbons and ligand, respectively (Fig. 7E,F). The minor fluctuations noted from the RMSD plot correspond to the dynamic movement of the compound **3** glutamic acid and loop 1 of MTHFD2L. The essential protein–ligand interactions were maintained similarly for compound **3** at the MTHFD2 binding site, as compared to compounds **1** and **2**; however, a few new and additional interactions were noted that are believed to altogether contribute to the binding affinity for MTHFD2L with an experimental IC_50_ value of 47 nM. The H-bond interactions between the diaminopyrimidine scaffold and Val145, Leu147 and Asp169 account for 80%, 51% and 100% of the simulation time, respectively (Fig. S35). The amide carbonyl and the backbone oxygen of **3** H-bond to Asn101 of MTHFD2L, for a total of 100% of the simulation time. The amide carbonyl near to the diaminopyrimidine ring forms H-bond contact with Lys102 for 70% of the simulation time. Interestingly, no π–π stacking is observed between the central 3-flurophenyl ring and Tyr98; instead the glutamic acid sidechain of **3** H-bonds to Tyr86 for 44% of the simulation trajectory. The flexible glutamic acid sidechain of **3** was furthermore able to constitute two new H-bond interactions: with Lys302 and Leu303 that were maintained for 26% and 32% of the simulation trajectory, respectively. The existence of these two interactions furthermore pinpoints the occupancy of compound **3** deeper into the substrate binding site of MTHFD2L, correlating with its experimental activity.

### Insights into the MTHFD loop1 conformational changes in presence of compounds 1–3

**Fig. 8:**
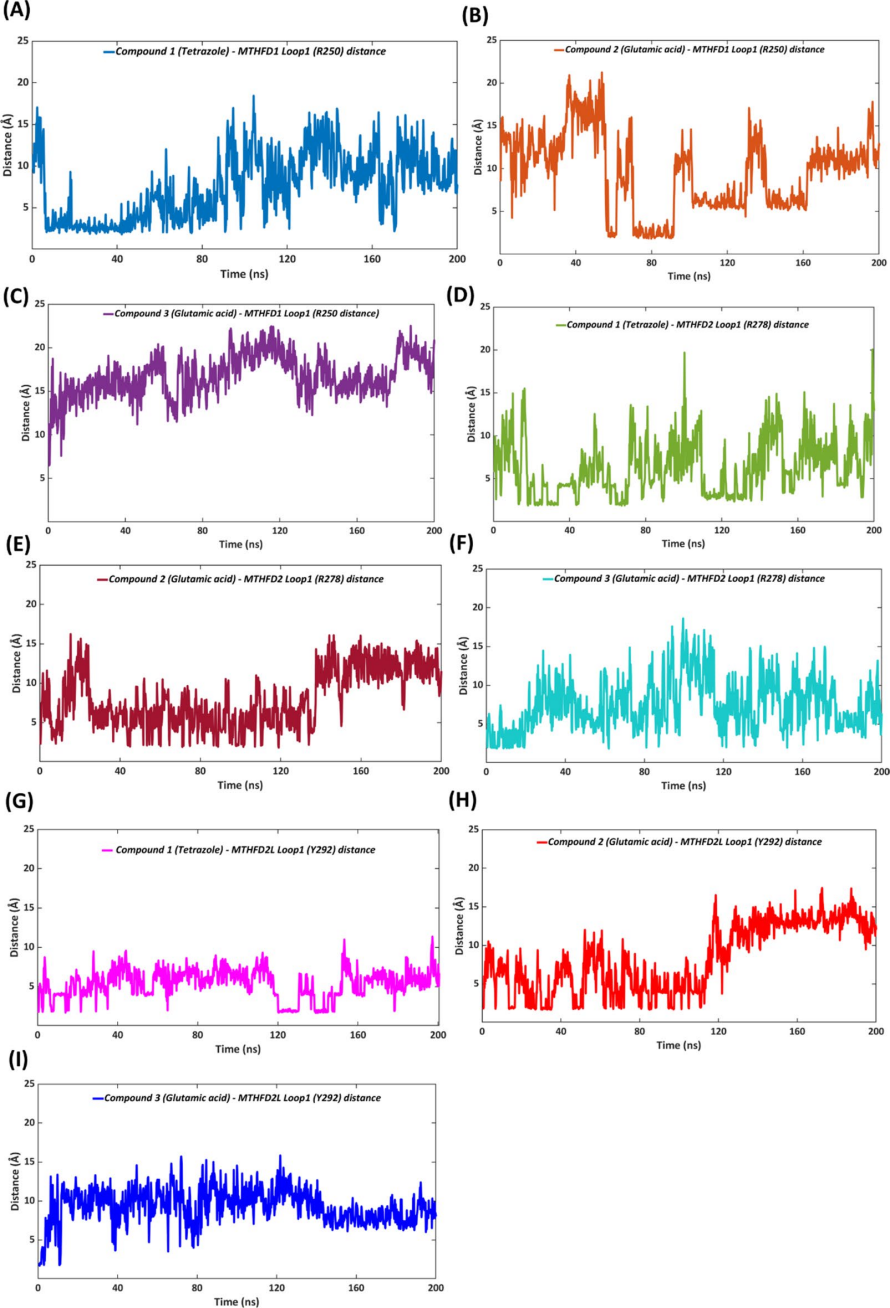
Distance of the tetrazole unit of **1** with: (**A**) Arg250 of MTHFD1 loop 1 (blue), (**D**) Arg278 of MTHFD2 loop 1 (green), and (**G**) Tyr292 of MTHFD2L loop 1 (magenta) over the course of 200 ns MD simulation. Distance of the glutamic acid unit of **2** with: (**B**) Arg250 of MTHFD1 loop 1 (orange), (**E**) Arg278 of MTHD2 loop 1 (brown), and (**H**) Tyr292 of MTHFD2L loop 1 (red). Distance of the glutamic acid unit of **3** with: (**C**) Arg250 of MTHFD1 loop 1 (violet), (**F**) Arg278 of MTHD2 loop 1 (cyan), and (**I**) Tyr292 of MTHFD2L loop 1 (dark blue).

In order to shed further light on loop 1 conformational changes of the MTHFD isoforms when the diaminopyrimidine-based inhibitors **1–3** are bound, we investigated the distance between these and loop 1 of MTHFD1, MTHFD2 and MTHFD2L during the course of the simulations. The information gathered shows a good correlation with the reported experimental activity. The average distance between the tetrazole unit of **1** and Arg250 of the MTHFD1 loop 1 during the simulation, was 7.50 Å (Table 3, Fig. 8A) which correlates with the observed loop 1 movement (Fig. 3C,D) and the maintenance of H-bond interaction between compound **1** and Arg250 for 36% of the simulation time, as discussed earlier, leading to the 0.5 nM MTHFD1 inhibitory activity of observed for **1**. Despite the H-bond contact between compound **2** and Arg250 of the MTHFD1 loop 1 that exists during 61% of the simulation time (as discussed earlier), limited movement of loop 1 was observed when **2** is bound. Moreover, the average distance between the glutamic acid unit of **2** and Arg250 of the MTHFD1 loop 1 was found to be 9.52 Å (Table 3, Fig. 8B) which denotes reduced proximity between **2** and the MTHFD1 loop 1, corresponding to the comparatively weaker MTHFD1 inhibitory activity with an IC_50_ of 89 nM. The comparative analysis furthermore verifies the fact that conformational changes are more favored when the tetrazole unit (compound **1**) rather than the glutamic acid moiety (compound **2**) is in the proximity of the MTHFD1 loop1.

**Table 3:** Average distance (in Å) between **1–3** and loop 1 of the MTHFD isoforms during the 200 ns MD simulations, along with their reported IC_50_ values.

Entry	MTHFD1		MTHFD2		MTHFD2L	
	Avg. distance (Å) with Loop 1 (Arg250)	IC_50_ (nM)	Avg. distance (Å) Loop 1 (Arg278)	IC_50_ (nM)	Avg. distance (Å) with Loop 1 (Tyr292)	IC_50_ (nM)
**1** (Tetrazole)	7.50	0.5	6.37	11	5.73	27
**2** (Glutamic acid)	9.52	89	7.97	254	8.31	126
**3** (Glutamic acid)	16.75	16	7.46	47	9.31	47

Compound **3** is characterized by the presence of a central 3-fluoropyridine ring whereas **1** and **2** have pyridine and phenyl rings at the same position, respectively. As mentioned above, compound **3** does not interact with loop 1 of MTHFD1, as evidenced by the 16.75 Å average distance between the glutamic acid of **3** and Arg250 (Table 3, Fig. 8C). However, two additional H-bond interactions (with Lys10 and Arg17) were observed which underline the placement of compound **3** much deeper into the substrate binding pocket of MTHFD1 and further substantiates the remarkably potent activity of **3** against MTHFD1 (IC_50_ = 16 nM). It can thus be hypothesized that the deeper accommodation of compound **3** in the substrate binding site of MTHFD1, anchored by Lys10 and Arg17 H-bond contacts, prevents the interaction of the glutamic acid unit with loop 1 of MTHFD1, and further improves the binding affinity.

Lys10 in MTHFD1 is replaced by Arg43 in MTHFD2 (Fig. 2B). Arg43 is more accessible to the diaminopyrimidine-based inhibitors due to the projection of its flexible guanidine group toward the substrate binding site, whereas Lys10 in MTHFD1 is oriented deeper into the substrate binding site. Arg17 in MTHFD1 is similarly replaced by Lys50 in MTHFD2. Compounds **1–3** all form H-bond interaction with Arg43 for 25–40% of the simulation time, demonstrating promising selectivity potential; however, presence of the diaminopyrimidine scaffold in compounds **1–3** limits the opportunities for MTHFD2 selectivity. Besides this, the tetrazole unit of **1** similarly H-bonds to Arg278 of MTHFD2 loop 1 for 63% of simulation time, correlating with an average distance of 6.37 Å (Table 3, Fig. 8D) and an 11 nM IC_50_ of compound **1** towards MTHFD2. The glutamic acid unit of compound **2** displays a larger average distance of 7.97 Å (Table 3, Fig. 8E) to Arg278 of the MTHFD2 loop 1, and a corresponding existence of just 17% of H-bond contact during the simulation. This agrees with the negligible contact of the glutamic acid of **2** with loop 1 of MTHFD2 and furthermore correlates with the relatively poor MTHFD2 inhibitory activity with an IC_50_ of 254 nM. The average distance between the glutamic acid unit of compound **3** and Arg278 was found to be 7.46 Å during the simulation (Table 3, Fig. 8F). Unlike the MTHFD1—compound **3** complex, the glutamic acid unit of **3** is limited by the H-bond contact with Arg43 and thus cannot access Lys50 (Arg17 in MTHFD1) that is placed much deeper into the substrate binding pocket. This is in turn compensated by the formation of H-bond interaction with Arg278 of loop 1 during 43% of the simulation time, further justifying the 47 nM MTHFD2 inhibitory activity of compound 3 (relative to its 16 nM MTHFD1 inhibitory activity).

In comparison with the MTHFD1 and MTHFD2 structures, the diaminopyrimidine-based inhibitors **1–3** show limited interaction with loop1 of MTHFD2L. The tetrazole unit of compound **1** was able to H-bond to Tyr292 of MTHFD2L loop1 for just 24% of the simulation time, however, the average distance of 5.73 Å (Table 3, Fig. 8G) indicates a closer proximity and reduced dynamic movement of the MTHFD2L loop1. Despite this, **1** displays an exceptional interaction profile with all essential residues at the substrate binding site of MTHFD2L which leads to its 27 nM potent inhibitory activity. The glutamic acid unit of compound **2** H-bonds to Tyr292 of the MTHFD2L loop 1 for 31% of the simulation time. The average distance between the two was calculated to be 8.31 Å over the course of the simulation (Table 3, Fig. 8H). Despite a reasonable contact with Tyr292 of the MTHFD2L loop 1, the higher average distance can be justified by the fact that the glutamic acid unit and the central phenyl ring of **2** undergo some peculiar conformational changes, as discussed earlier, which in turn affect the dynamic movement of α-helix 1 of MTHFD2L (Fig. S15), establishing a reasonable correlation with the weaker MTHFD2L inhibitory activity observed (IC_50_ = 126 nM). The glutamic acid unit of **3**, finally, showed no interactions with loop 1 of MTHFD2L which correlates with the average distance of 9.31 Å during the course of the simulation (Table 3, Fig. 8I). Apart from maintaining essential protein–ligand contacts at the substrate binding site of MTHFD2L, two new interactions were observed (with Lys302 and Leu303) that enable the 47 nM inhibitory activity of compound **3** against MTHFD2L.

### Binding free energy calculations

In order to further analyse and validate the co-crystallized/docking poses of diaminopyrimidine-based inhibitors with respect to the three MTHFD isoforms, we performed binding free energy calculations using the MM-GBSA method. The binding free energies (ΔG Bind), the contributions from H-bond energy (ΔG Hbond), Lipophilic energy (ΔG Lipophilic) and van der Waals energy (ΔG van der Waals) of compounds 1–3 were computed and enlisted in Tables 4, 5 and 6 for MTHFD1, MTHFD2 and MTHFD2L, respectively, along with the reported IC_50_ values. ΔG Bind of all compounds ranges from − 65.08 to − 89.94 kcal/mol, which confirm non-selective binding across the three isoforms, further correlating with their nanomolar potent inhibition. Similarly, the ΔG Hbond lies between − 6.35 and − 10.89 kcal/mol among diaminopyrimidine-based inhibitors, indicating occurrence of a reasonable number of H-bonding interactions between the ligand and the protein, as also discussed from the aforementioned docking and MD analyses. In particular, the substrate site residues Lys56, Val99, Gln100, Leu101, Asp125 (MTHFD1), Lys88, Val131, Gln132, Leu133, Asp155 (MTHFD2) and Lys102, Val145, Gln146, Leu147, Asp169 (MTHFD2L) are largely involved in H-bond interactions with the diaminopyrimidine scaffold of compounds 1–3, leading to non-selective inhibition. The hydrophobic residue Tyr52 (MTHFD1)/Tyr84 (MTHFD2)/Tyr98 (MTHFD2L) constitutes π–π stacking with the phenyl/pyridyl ring system of compounds 1–3, while the longer and bulkier Arg250 (MTHFD1 loop1)/Arg278 (MTHFD2 loop1)/Tyr292 (MTHFD2L loop1) is associated with the conformational changes when compounds 1–3 are bound via van der Waals /H-bond interactions. The contribution of thus ΔG Lipophilic and ΔG van der Waals is a key factor in improving the binding affinity between the inhibitors and the proteins. ΔG Lipophilic ranges from − 11.37 to − 13.14 kcal/mol, whereas ΔG van der Waals has the largest contribution among all other energetic terms, ranging between − 53.46 and − 66.84 kcal/mol. The overall energy contributions of each inhibitor against the MTHFD isoforms thus corroborate with the reported experimental IC_50_ values_,_ confirming the non-selective behaviour, as all the energetic terms are in close range. With the purpose of further getting insights of selectivity, we compared the binding mode of one of the non-selective diaminopyrimidine-based inhibitors (compound 1) against the selective coumarin-based inhibitor (compound 5) at the substrate binding site of MTHFD2, as following.

**Table 4:** MM-GBSA binding free energy results (ΔG bind, ΔG Hbond, ΔG lipophilic and ΔG van der Waals in kcal/mol) of compounds **1–3** in MTHFD1.

Entry	ΔG bind	ΔG Hbond	ΔG lipophilic	ΔG van der Waals	IC_50_ (nM)
Compound 1	− 65.08	− 7.18	− 11.55	− 63.31	0.5
Compound 2	− 61.67	− 6.35	− 12.20	− 57.99	89
Compound 3	− 65.07	− 7.03	− 12.87	− 59.35	16

**Table 5:** MM-GBSA binding free energy results (ΔG bind, ΔG Hbond, ΔG lipophilic and ΔG van der Waals in kcal/mol) of compounds **1–3** in MTHFD2.

Entry	ΔG bind	ΔG Hbond	ΔG lipophilic	ΔG van der Waals	IC_50_ (nM)
Compound 1	− 79.46	− 7.50	− 13.14	− 66.84	11
Compound 2	− 72.03	− 7.27	− 11.61	− 54.78	254
Compound 3	− 81.78	− 7.48	− 11.60	− 61.02	47

**Table 6:** MM-GBSA binding free energy results (ΔG bind, ΔG Hbond, ΔG lipophilic and ΔG van der Waals in kcal/mol) of compounds **1–3** in MTHFD2L.

Entry	ΔG bind	ΔG Hbond	ΔG lipophilic	ΔG van der Waals	IC_50_ (nM)
Compound 1	− 80.47	− 10.43	− 11.37	− 62.42	27
Compound 2	− 88.24	− 10.89	− 12.62	− 60.31	126
Compound 3	− 89.94	− 10.27	− 12.13	− 53.46	47

### Selective coumarin-based inhibitor vs non-selective diaminopyrimidine-based inhibitor

As mentioned in the introduction section, the two classes of MTHFD2 inhibitors, the diaminopyrimidine-based compounds (**1–3**)^5^ and the tricyclic coumarin-based compounds (**4–6**)^22^ bind to the substrate binding site of MTHFD2. The diaminopyrimidine-based inhibitors bind non-selectively whereas the tricyclic coumarin-based inhibitors bind selectively to MTHFD2. We tried to extend our insights on the aforementioned selectivity preferences and differences at the substrate binding site level involving the two classes of MTHFD2 inhibitors. Taking compounds **1** and **5** as examples, the diaminopyrimidine-based inhibitor **1** has demonstrated IC_50_ values of 0.5 nM and 11 nM against MTHFD1 and MTHFD2L isoforms^5^, respectively, confirming the non-selective mode of MTHFD2 inhibition, whereas the tricyclic coumarin-based inhibitor **5** has shown IC_50_ values of 6.4 µM and 48 nM against MTHFD1 and MTHFD2, respectively, confirming the selective mode of MTHFD2 inhibition^22^. We thus superposed the X-ray structure of MTHFD2 in complex with **1** (PDB code: 6S4A) with the X-ray structure of MTHFD2 bound to **5** (PDB code: 6KG2).

**Fig. 9:**
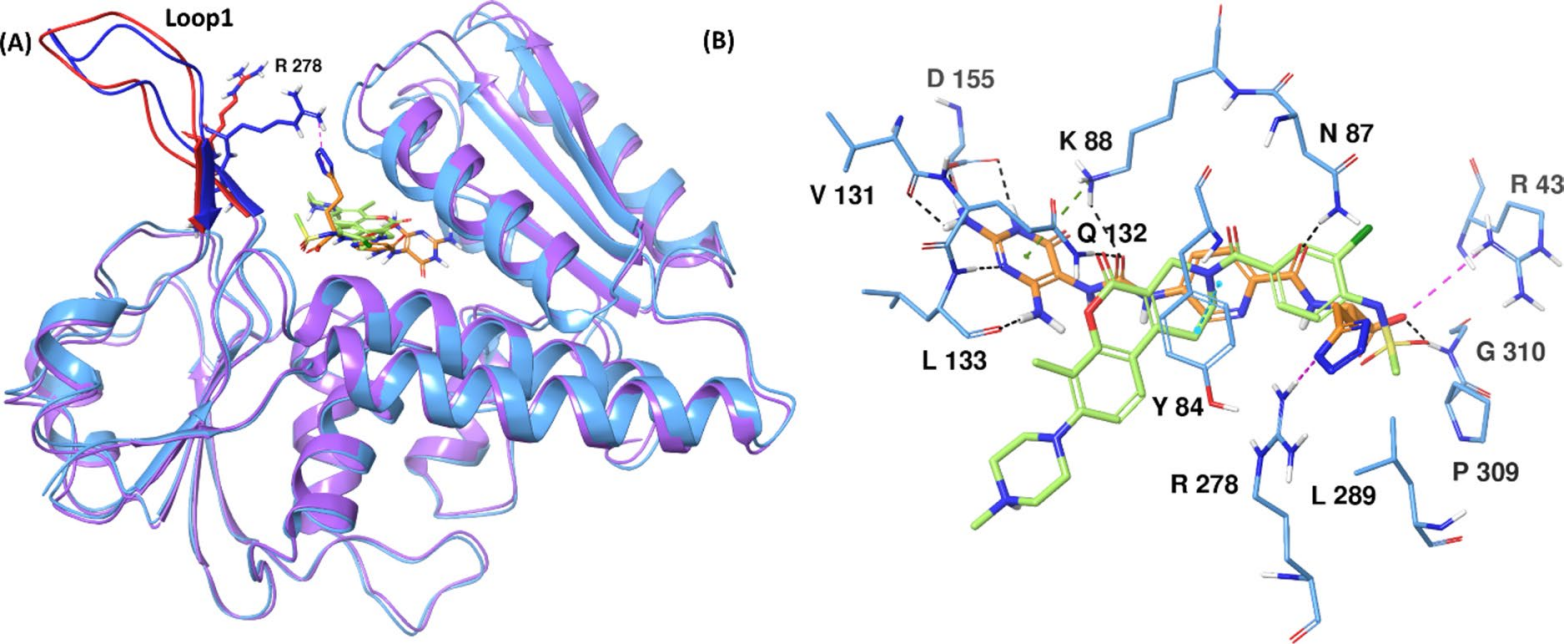
(**A**) Ribbon view: X-ray structure of MTHFD2 in complex with diaminopyrimidine-based inhibitor **1** (protein ribbons in blue, loop 1 in dark blue, compound **1** in orange, PDB code: 6S4A), superposed with the X-ray structure of MTHFD2 in complex with tricyclic coumarin-based inhibitor **5** (protein ribbons in purple, loop 1 in red, compound **5** in light green, PDB code: 6KG2). (**B**) Binding site view: compound **1** in orange, **5** in light green and the MTHFD2 residues (6S4A) in blue.

As shown in Fig. 9A, the tetrazole unit of **1** (supported by the butyl linker) is accessible to the MTHFD2 loop 1 forming H-bond/salt-bridge interactions with Arg278. The same tetrazole-butyl unit is also in close proximity of loop 1 of the MTHFD1 and MTHFD2L isoforms, capable of forming H-bond/salt-bridge interactions with Tyr240, Arg250 (MTHFD1) and Tyr292 (MTHFD2L). Compound **5**, on the other hand, is characterized by the presence of a terminal *N*-(2-chlorophenyl)methanesulfonamide group at the same position, which is a significantly smaller and less flexible group relative to the tetrazole-butyl unit of **1**. The *N*-(2-chlorophenyl)methanesulfonamide of **5** is not accessible to loop 1 of MTHFD2 (and correspondingly in MTHFD1 and MTHFD2L) and is limited to interacting only with the substrate site residues, forming H-bond/salt-bridge contacts with Arg43 and Gly310 that play key roles in the MTHFD2 selectivity (Fig. 9B)^24^. Despite the high nanomolar activities, the incorporation of longer and flexible moieties such as tetrazole-butyl (compound **1**) and glutamic acid (compounds **2–3**) at the termini are therefore not recommended when designing selective MTHFD2 inhibitors, in order to limit the interactions with and/or conformational changes of loop 1 in all three MTHFD isoforms. Instead smaller scaffolds at the same position such as benzoic acid (compound **4**) and *N*-(2-chlorophenyl)methanesulfonamide (compound **5**) restrict the binding to only the substrate site residues that are important for selectivity.

Another significant difference between the binding modes of **1** and **5** involves the presence of the diaminopyrimidine ring (compound **1**) which extends deeper into the substrate binding site of MTHFD2 forming multiple H-bond interactions as compared to the solvent exposed *N*-methylpiperazine linked to the tricyclic coumarin ring system of compound **5**. The diaminopyrimidine scaffold is proven to be an important structural element in improving the binding affinity for MTHFD2 as it engages Lys88, Val131, Leu133 and Asp155 via H-bond interactions. However, these residues are identical in the other two MTHFD isoforms (Lys56, Val99, Leu101, Asp125 in MTHFD1, and Lys102, Val145, Leu147, Asp169 in MTHFD2L), further narrowing the possibilities of selective MTHFD2 inhibition with compounds bearing the diaminopyrimidine-based scaffold. In contrast to this, compound **5** comprises of the solvent exposed *N*-methylpiperazine ring which significantly improves the solubility and pharmacokinetic properties, and further broadens the scope of selective MTHFD2 inhibition as it does not engage the aforementioned substrate site residues of either MTHFD isoform. Moreover, compound **4** has a solvent-exposed tricyclic coumarin ring system as it lacks any aliphatic monocyclic ring such as the *N*-methylpiperazine of **5**. Despite that the binding affinity of **4** for MTHFD2 was reduced to an IC_50_ of 1.6 μM, it was able to show > 18-fold selectivity for MTHFD2^22^. The information gathered on correlating the binding mode and the enzyme activities of compounds **4–5** against MTHFD2 signifies how the solvent-exposed aliphatic monocyclic systems and the central tricyclic coumarin-based scaffold play pivotal roles in facilitating selectivity, and should thus be taken into consideration when developing new and selective MTHFD2 inhibitors.

## Conclusions

In the present work, extensive computational modeling involving molecular docking and molecular dynamics simulations has been carried out on the diaminopyrimidine-based inhibitors (compounds **1–3**) bound to MTHFD2. These diaminopyrimidine-based compounds were reported as highly potent inhibitors of MTHFD2 showing notable anticancer activities both in vitro and in vivo^5^. Challengingly, they also demonstrated strong inhibition of the MTHFD1 and MTHFD2L isoforms, which are expressed in healthy adult human tissues, unlike MTHFD2 that is explicitly expressed in several cancer cells. We first superposed the available X-ray structures of MTHFD1, MTHFD2 and MTHFD2L and compared their substrate binding sites when the diaminopyrimidine-based inhibitor **1** is bound, in order to understand the non-selective mode of binding. An in-depth analysis of the binding modes of **1–3** with respect to all three isoforms MTHFD1, MTHFD2 and MTHFD2L was carried out using docking and MD simulations, which allowed us to gain useful insights on protein conformational changes (loop 1 in particular) and dynamic movements of the compounds, further establishing a good correlation with the reported experimental activities. In general, loop 1 of the MTHFD isoforms displayed notable movement and conformational flexibility in presence of compounds **1–3**; in particular the tetrazole unit of **1** seems to be favored over the glutamic acid unit of compounds **2–3** by interacting with Arg250 (MTHFD1), Arg278 (MTHFD2) and Tyr292 (MTHFD2L) of loop 1. The interaction of compounds **1–3** with the MTHFD loop 1 and the associated conformational changes seem to be desirable for the MTHFD activity and thus crucially contribute to the potent nanomolar binding affinities. However, this also gives rise to selectivity issues suggesting that the longer and flexible structural elements in the diaminopyrimidine-based inhibitors such as the tetrazole-butyl and glutamic acid units are not favorable due to their strong interaction also with loop 1 of MTHFD1 and MTHFD2L. Loop1 of MTHFD2L seems to fluctuate less in presence of the diaminopyrimidine-based compounds, as compared to loop 1 of MTHFD1 and MTHFD2. Moreover, the incorporation of the central 3-fluropyridine ring system in **3** favors its glutamic acid unit to bind deeper into the substrate binding pocket of MTHFD1 and MTHFD2L (rather than interacting with loop 1) and form additional H-bond interactions with Lys10, Arg 17 (MTHFD1) and Lys302, Leu303 (MTHFD2L). Either the deep penetration into the substrate binding site of MTHFD isoforms or the interactions with the MTHFD loop 1, inducing further conformational changes, appear to be crucial for the improved binding affinities of **1–3**.

We also superposed and compared the co-crystallized pose of the non-selective diaminopyrimidine-based inhibitor **1** with the co-crystallized pose of selective tricyclic coumarin-based inhibitor **5**^22^ in order to understand the selectivity preferences and differences. As mentioned above, the diaminopyrimidine-based inhibitors have an extended tetrazole-butyl/glutamic acid unit which interacts with the MTHFD loop 1. The tricyclic coumarin-based inhibitor **5** instead has an *N*-(2-chlorophenyl)methanesulfonamide moiety that is not accessible to the MTHFD loop 1 and is limited to the substrate binding pocket only. To this end, the relatively smaller groups such as *N*-(2-chlorophenyl)methanesulfonamide should thus be favoured over extensions such as tetrazole-butyl and glutamic acid for MTHFD2 selectivity. Despite that the diaminopyrimidine scaffold of compounds **1–3** is proven to be an important structural element in facilitating nanomolar activity, it also leads to non-selective mode of binding forming identical interactions in the MTHFD substrate binding sites. The tricyclic coumarin-based compound **5** instead has an *N*-methylpiperazine ring system that is solvent exposed and contribute to solubility and pharmacokinetic properties^22^. The aliphatic monocyclic ring system thus appears to be favoured over aromatic heterocycles such as the diaminopyrimidine ring systems to attain MTHFD2 selectivity. The authors believe that the findings from this extensive computational modeling will provide enhanced understanding in addressing the selectivity issues of the proposed diaminopyrimidine-based MTHFD2 inhibitors. Furthermore, the in-depth analysis of the binding modes of diaminopyrimidine-based compounds will guide medicinal chemists and researchers in the rational design of new and selective MTHFD2 inhibitors by avoiding incorporation of structural elements that do not favour MTHFD2 selectivity.

## Supplementary Information

Supplementary Information.

## Data Availability

The structures of the docked complexes, and the MD trajectory files of compounds **1–3** with respect to MTHFD1, MTHFD2 and MTHFD2L, are provided as tarballs (.tar.gz) freely accessible at zenodo.org: 10.5281/zenodo.8382373.

## References

[1] Gustafsson, R. *et al.* Crystal structure of the emerging cancer target MTHFD2 in complex with a substrate-based inhibitor. *Cancer Res.* **77**, 937–948 (2017). 27899380 10.1158/0008-5472.CAN-16-1476

[2] Liu, F. *et al.* Increased MTHFD2 expression is associated with poor prognosis in breast cancer. *Tumor Biol.* **35**, 8685–8690 (2014). 10.1007/s13277-014-2111-x 24870594

[3] Ju, H.-Q. *et al.* Modulation of redox homeostasis by inhibition of MTHFD2 in colorectal cancer: Mechanisms and therapeutic implications. *J. Natl. Cancer Inst.* **111**, 584–596 (2019). 30534944 10.1093/jnci/djy160 PMC6579745

[4] Pikman, Y. *et al.* Targeting MTHFD2 in acute myeloid leukemia. *J. Exp. Med.* **213**, 1285–1306 (2016). 27325891 10.1084/jem.20151574 PMC4925018

[5] Bonagas, N. *et al.* Pharmacological targeting of MTHFD2 suppresses acute myeloid leukemia by inducing thymidine depletion and replication stress. *Nat. Cancer* **3**, 156–172 (2022). 35228749 10.1038/s43018-022-00331-y PMC8885417

[6] Lin, H. *et al.* MTHFD2 overexpression predicts poor prognosis in renal cell carcinoma and is associated with cell proliferation and vimentin-modulated migration and invasion. *Cell. Physiol. Biochem.* **51**, 991–1000 (2018). 30466107 10.1159/000495402

[7] Liu, X. *et al.* Methylenetetrahydrofolate dehydrogenase 2 overexpression is associated with tumor aggressiveness and poor prognosis in hepatocellular carcinoma. *Dig. Liver Dis.* **48**, 953–960 (2016). 27257051 10.1016/j.dld.2016.04.015

[8] Andrew, A. S. *et al.* Bladder cancer SNP panel predicts susceptibility and survival. *Hum. Genet.* **125**, 527–539 (2009). 19252927 10.1007/s00439-009-0645-6 PMC2763504

[9] Ben-Sahra, I., Hoxhaj, G., Ricoult, S. J. H., Asara, J. M. & Manning, B. D. mTORC1 induces purine synthesis through control of the mitochondrial tetrahydrofolate cycle. *Science* **351**, 728–733 (2016). 26912861 10.1126/science.aad0489 PMC4786372

[10] Yu, C. *et al.* Down-regulation of MTHFD2 inhibits NSCLC progression by suppressing cycle-related genes. *J. Cell. Mol. Med.* **24**, 1568–1577 (2020). 31778025 10.1111/jcmm.14844 PMC6991687

[11] Koufaris, C. *et al.* Suppression of MTHFD2 in MCF-7 breast cancer cells increases glycolysis, dependency on exogenous glycine, and sensitivity to folate depletion. *J. Proteome Res.* **15**, 2618–2625 (2016). 27315223 10.1021/acs.jproteome.6b00188

[12] Emmanuel, N. *et al.* Purine nucleotide availability regulates mTORC1 activity through the Rheb GTPase. *Cell Rep.* **19**, 2665–2680 (2017). 28658616 10.1016/j.celrep.2017.05.043

[13] Shang, M. *et al.* The folate cycle enzyme MTHFD2 induces cancer immune evasion through PD-L1 up-regulation. *Nat. Commun.* **12**, 1940 (2021). 33782411 10.1038/s41467-021-22173-5 PMC8007798

[14] Shi, Y. *et al.* MTHFD2 promotes tumorigenesis and metastasis in lung adenocarcinoma by regulating AKT/GSK-3β/β-catenin signalling. *J. Cell. Mol. Med.* **25**, 7013–7027 (2021). 34121323 10.1111/jcmm.16715 PMC8278097

[15] Koufaris, C. & Nilsson, R. Protein interaction and functional data indicate MTHFD2 involvement in RNA processing and translation. *Cancer Metab.* **6**, 12 (2018). 30275950 10.1186/s40170-018-0185-4 PMC6158883

[16] Green, N. H. *et al.* MTHFD2 links RNA methylation to metabolic reprogramming in renal cell carcinoma. *Oncogene* **38**, 6211–6225 (2019). 31289360 10.1038/s41388-019-0869-4 PMC8040069

[17] Gustafsson Sheppard, N. *et al.* The folate-coupled enzyme MTHFD2 is a nuclear protein and promotes cell proliferation. *Sci. Rep.* **5**, 15029 (2015). 26461067 10.1038/srep15029 PMC4602236

[18] Tedeschi, P. M., Vazquez, A., Kerrigan, J. E. & Bertino, J. R. Mitochondrial methylenetetrahydrofolate dehydrogenase (MTHFD2) overexpression is associated with tumor cell proliferation and is a novel target for drug development. *Mol. Cancer Res.* **13**, 1361–1366 (2015). 26101208 10.1158/1541-7786.MCR-15-0117 PMC4618031

[19] Scaletti, E. R. *et al.* The first structure of human MTHFD2L and its implications for the development of isoform-selective inhibitors. *ChemMedChem* **17**, e202200274 (2022). 35712863 10.1002/cmdc.202200274 PMC9796130

[20] Fu, C. *et al.* The natural product carolacton inhibits folate-dependent C1 metabolism by targeting FolD/MTHFD. *Nat. Commun.* **8**, 1529 (2017). 29142318 10.1038/s41467-017-01671-5 PMC5688156

[21] Kawai, J. *et al.* Structure-based design and synthesis of an isozyme-selective MTHFD2 inhibitor with a tricyclic coumarin scaffold. *ACS Med. Chem. Lett.* **10**, 893–898 (2019). 31223444 10.1021/acsmedchemlett.9b00069 PMC6580548

[22] Kawai, J. *et al.* Discovery of a potent, selective, and orally available MTHFD2 inhibitor (DS18561882) with in vivo antitumor activity. *J. Med. Chem.* **62**, 10204–10220 (2019). 31638799 10.1021/acs.jmedchem.9b01113

[23] Lee, L.-C. *et al.* Xanthine derivatives reveal an allosteric binding site in methylenetetrahydrofolate dehydrogenase 2 (MTHFD2). *J. Med. Chem.* **64**, 11288–11301 (2021). 34337952 10.1021/acs.jmedchem.1c00663 PMC8389891

[24] Jha, V., Holmelin, F. L. & Eriksson, L. A. Binding analysis and structure-based design of tricyclic coumarin-derived MTHFD2 inhibitors as anticancer agents: Insights from computational modeling. *ACS Omega* **8**, 14440–14458 (2023). 37125100 10.1021/acsomega.2c08025 PMC10134251

[25] Jha, V. & Eriksson, L. A. Binding modes of xanthine-derived selective allosteric site inhibitors of MTHFD2. *ChemistryOpen* **12**, e202300052 (2023). 37129313 10.1002/open.202300052 PMC10152887

[26] Bueno, R., Dawson, A. & Hunter, W. N. An assessment of three human methylenetetrahydrofolate dehydrogenase/cyclohydrolase-ligand complexes following further refinement. *Acta Crystallogr. Sect. F* **75**, 148–152 (2019). 10.1107/S2053230X18018083 PMC6404858 30839287

[27] Berman, H. M. *et al.* The protein data bank. *Nucleic Acids Res.* **28**, 235–242 (2000). 10592235 10.1093/nar/28.1.235 PMC102472

[28] Madhavi Sastry, G., Adzhigirey, M., Day, T., Annabhimoju, R. & Sherman, W. Protein and ligand preparation: Parameters, protocols, and influence on virtual screening enrichments. *J. Comput. Aided. Mol. Des.* **27**, 221–234 (2013). 23579614 10.1007/s10822-013-9644-8

[29] *Schrödinger Release 2023-2: Maestro* (Schrödinger, LLC, 2023).

[30] Jacobson, M. P. *et al.* A hierarchical approach to all-atom protein loop prediction. *Proteins Struct. Funct. Bioinform.* **55**, 351–367 (2004). 10.1002/prot.10613 15048827

[31] Jacobson, M. P., Friesner, R. A., Xiang, Z. & Honig, B. On the role of the crystal environment in determining protein side-chain conformations. *J. Mol. Biol.* **320**, 597–608 (2002). 12096912 10.1016/S0022-2836(02)00470-9

[32] *Schrödinger Release 2023-2: Prime* (Schrödinger, LLC, 2023).

[33] Olsson, M. H. M., Søndergaard, C. R., Rostkowski, M. & Jensen, J. H. PROPKA3: Consistent treatment of internal and surface residues in empirical pKa predictions. *J. Chem. Theory Comput.* **7**, 525–537 (2011). 26596171 10.1021/ct100578z

[34] Lu, C. *et al.* OPLS4: Improving force field accuracy on challenging regimes of chemical space. *J. Chem. Theory Comput.* **17**, 4291–4300 (2021). 34096718 10.1021/acs.jctc.1c00302

[35] Jorgensen, W. L. & Tirado-Rives, J. The OPLS [optimized potentials for liquid simulations] potential functions for proteins, energy minimizations for crystals of cyclic peptides and crambin. *J. Am. Chem. Soc.* **110**, 1657–1666 (1988). 27557051 10.1021/ja00214a001

[36] Jorgensen, W. L., Maxwell, D. S. & Tirado-Rives, J. Development and testing of the OPLS all-atom force field on conformational energetics and properties of organic liquids. *J. Am. Chem. Soc.* **118**, 11225–11236 (1996). 10.1021/ja9621760

[37] Shivakumar, D. *et al.* Prediction of absolute solvation free energies using molecular dynamics free energy perturbation and the OPLS force field. *J. Chem. Theory Comput.* **6**, 1509–1519 (2010). 26615687 10.1021/ct900587b

[38] Shelley, J. C. *et al.* Epik: A software program for pKa prediction and protonation state generation for drug-like molecules. *J. Comput. Aided. Mol. Des.* **21**, 681–691 (2007). 17899391 10.1007/s10822-007-9133-z

[39] Greenwood, J. R., Calkins, D., Sullivan, A. P. & Shelley, J. C. Towards the comprehensive, rapid, and accurate prediction of the favorable tautomeric states of drug-like molecules in aqueous solution. *J. Comput. Aided. Mol. Des.* **24**, 591–604 (2010). 20354892 10.1007/s10822-010-9349-1

[40] Friesner, R. A. *et al.* Glide: A new approach for rapid, accurate docking and scoring. 1. Method and assessment of docking accuracy. *J. Med. Chem.* **47**, 1739–1749 (2004). 15027865 10.1021/jm0306430

[41] Halgren, T. A. *et al.* Glide: A new approach for rapid, accurate docking and scoring. 2. Enrichment factors in database screening. *J. Med. Chem.* **47**, 1750–1759 (2004). 15027866 10.1021/jm030644s

[42] Bowers, K. J. *et al.* Scalable algorithms for molecular dynamics simulations on commodity clusters. In *SC’06: Proceedings of the 2006 ACM/IEEE Conference on Supercomputing* 43 (2006).

[43] Martyna, G. J., Klein, M. L. & Tuckerman, M. Nosé-Hoover chains: The canonical ensemble via continuous dynamics. *J. Chem. Phys.* **97**, 2635–2643 (1992). 10.1063/1.463940

[44] Wentzcovitch, R. M. Invariant molecular-dynamics approach to structural phase transitions. *Phys. Rev. B* **44**, 2358–2361 (1991). 10.1103/PhysRevB.44.2358 9999791

[45] Nosé, S. A unified formulation of the constant temperature molecular dynamics methods. *J. Chem. Phys.* **81**, 511–519 (1984). 10.1063/1.447334

