# Supplementary material for: Selectivity analysis of diaminopyrimidine-based inhibitors of MTHFD1, MTHFD2 and MTHFD2L
Source: Sci Rep. 2024 Sep 10;14:21073. doi: 10.1038/s41598-024-71879-1 (PMC11387627; doi:10.1038/s41598-024-71879-1)

**Supplementary Material**

# Selectivity analysis of diaminopyrimidine-based inhibitors of MTHFD1, MTHFD2 and MTHFD2L

Vibhu Jha[1,2], and Leif A. Eriksson[1*]

[1]Department of Chemistry and Molecular Biology, University of Gothenburg, 405 30 Göteborg, Sweden

[2]Institute of Cancer Therapeutics, School of Pharmacy and Medical Sciences, Faculty of Life Sciences, University of Bradford, Bradford, UK BD71DP

Correspondence:

*Leif A. Eriksson, Department of Chemistry and Molecular Biology,
University of Gothenburg, 405 30 Göteborg, Sweden.
Email: leif.eriksson@chem.gu.se
Phone: +46 317869117

## Table of contents

**Figure S1.** 2D structures of LYS345899, carolacton, HTS Hit and compound 10. P5

**Figure S2.** Docking poses of compounds **1-3** in the MTHFD1 binding site. P5

**Figure S3.** 2D Protein-ligand interaction diagram from the docking pose of MTHFD1 – compound 1. P6

**Figure S4.** 2D Protein-ligand interaction diagram of from the docking pose MTHFD1 – compound 2. P6

**Figure S5.** 2D Protein-ligand interaction diagram from the docking pose of MTHFD1 – compound 3. P7

**Figure S6.** RMSD analysis of the MTHFD1 – compound **1** complex from the triplicate MD simulations. P6

**Table S1.** Average RMSD values for the MTHFD1 – compound **1** complex from the triplicate MD simulations. P6

**Figure S7.** Protein-ligand interaction histogram from the MD simulations of compound **1** in the MTHFD1 binding site. P6

**Figure S8.** RMSD analysis of the MTHFD1 – compound **1** complex, showing protein conformational changes. P7

**Figure S9.** Superposed MD structure of theMTHFD1 – compound **1** complex with the X-ray structure of MTHFD1 without the substrate site inhibitor. P7

**Figure S10.** RMSD analysis of the MTHFD1 – compound **2** complex from the triplicate MD simulations. P8

**Table S2.** Average RMSD values for the MTHFD1 – compound **2** complex from the triplicate MD simulations. P8

**Figure S11.** MD snapshots of the MTHFD1 – compound **2** complex at 0 ns, 41 ns and 80 ns, showing conformational changes at the loop 1. P9

**Figure S12.** Protein-ligand interaction histogram from the MD simulations of compound **2** in the MTHFD1 binding site. P10

**Figure S13.** RMSD analysis of the MTHFD1 – compound **3** complex from the triplicate MD simulations. P10

**Table S3.** Average RMSD values for the MTHFD1 – compound **3** complex from the triplicate MD simulations. P10

**Figure S14.** Protein-ligand interaction histogram from the MD simulations of compound **3** in the MTHFD1 binding site. P11

**Figure S15.** RMSD analysis of the MTHFD2 – compound **1** complex from the triplicate MD simulations. P11

**Table S4.** Average RMSD values for the MTHFD2 – compound **1** complex from the triplicate MD simulations. P11

**Figure S16.** Cocrystallized poses of compounds **1-3** with MTHFD2. P13

**Figure S17.** 2D Protein-ligand interaction diagram from the co-crystallized pose of MTHFD2 – compound 1. P14

**Figure S18.** 2D Protein-ligand interaction diagram from the co-crystallized pose of MTHFD2 – compound 2. P14

**Figure S19.** 2D Protein-ligand interaction diagram from the co-crystallized pose of MTHFD2 – compound 3. P15

**Figure S20.** Protein-ligand interaction histogram from the MD simulations of compound **1** in the MTHFD2 binding site. P13

**Figure S21.** RMSD analysis of the MTHFD2 – compound **2** complex from the triplicate MD simulations. P13

**Table S5.** Average RMSD values for the MTHFD2 – compound **2** complex from the triplicate MD simulations. P13

**Figure S22.** Protein-ligand interaction histogram from the MD simulations of compound **2** in the MTHFD2 binding site. P14

**Figure S23.** RMSD analysis of the MTHFD2 – compound **3** complex from the triplicate MD simulations. P14

**Table S6.** Average RMSD values for the MTHFD2 – compound **3** complex from the triplicate MD simulations. P14

**Figure S24.** Protein-ligand interaction histogram from the MD simulations of compound **3** in the MTHFD2 binding site. P15

**Figure S25.** Docking/co-crystallized poses of compounds **1-3** in MTHFD2L. P15

**Figure S26.** 2D Protein-ligand interaction diagram from the docking pose of MTHFD2L – compound 1. P22

**Figure S27.** 2D Protein-ligand interaction diagram from the co-crystallized pose of MTHFD2L – compound 2. P23

**Figure S28.** 2D Protein-ligand interaction diagram from the docking pose of MTHFD2L – compound 3. P23

**Figure S29.** RMSD analysis of the MTHFD2L – compound **1** complex from the triplicate MD simulations. P16

**Table S7.** Average RMSD values for the MTHFD2L – compound **1** complex from the triplicate MD simulations. P16

**Figure S30.** Protein-ligand interaction histogram from the MD simulations of compound **1** in the MTHFD2L binding site. P16

**Figure S31.** RMSD analysis of the MTHFD2L – compound **2** complex from the triplicate MD simulations. P17

**Table S8.** Average RMSD values for the MTHFD2L – compound **2** complex from the triplicate MD simulations. P17

**Figure S32.** MD snapshots of the MTHFD2L – compound **2** complex at 104 ns and 120 ns, showing loop 1 and α-helix 1 conformational changes P17

**Figure S33.** Protein-ligand interaction histogram from the MD simulations of compound **2** in the MTHFD2L binding site. P18

**Figure S34.** RMSD analysis of the MTHFD2L – compound **3** complex from the triplicate MD simulations. P18

**Table S9.** Average RMSD values for the MTHFD2L – compound **3** complex from the triplicate MD simulations. P18

**Figure S35.** Protein-ligand interaction histogram from the MD simulations of compound **3** in the MTHFD2L binding site. P19

**Figure S1.** 2D structures of LYS345899, carolacton, HTS Hit and compound 10.

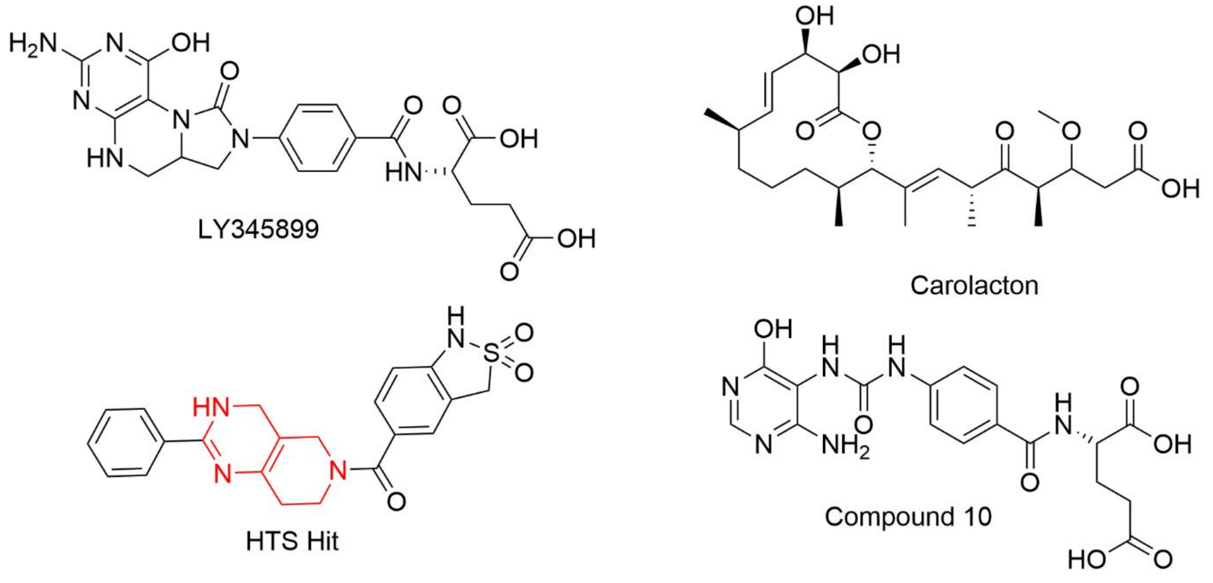


**Figure S2.** **(A)** Docking pose of compound **1** in the MTHFD1 binding site (ligand in orange, protein residues in pink). **(B)** Docking pose of compound **2** in the MTHFD1 binding site (ligand in green, protein residues in pink). **(C)** Docking pose of compound **3** in the MTHFD1 binding site (ligand in red, protein residues in pink).

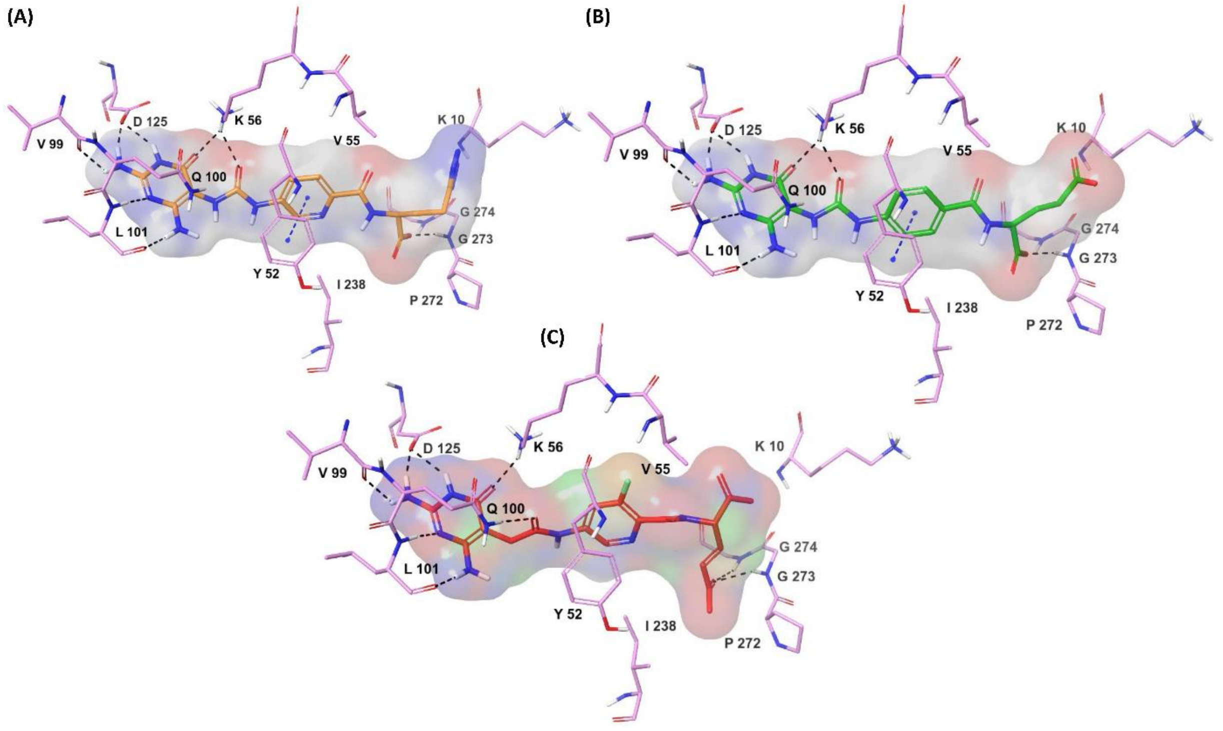

**Figure S3.** 2D Protein-ligand interaction diagram from the docking pose of MTHFD1 – compound 1.

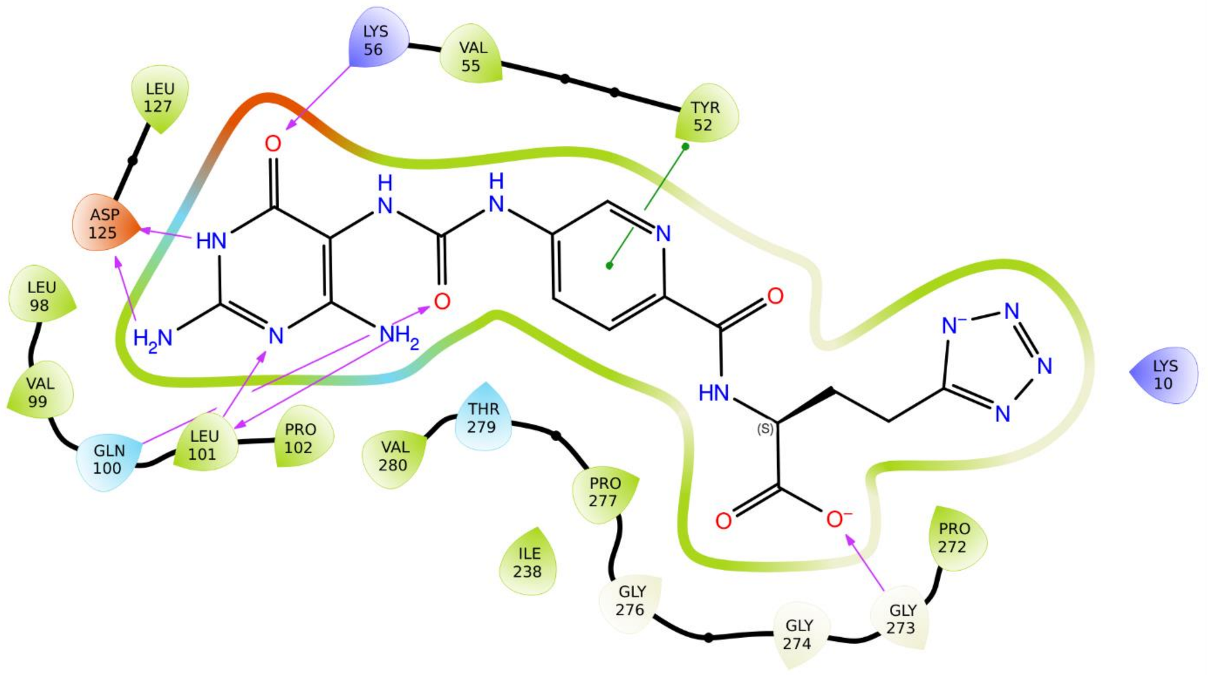


**Figure S4.** 2D Protein-ligand interaction diagram of from the docking pose MTHFD1 – compound 2.

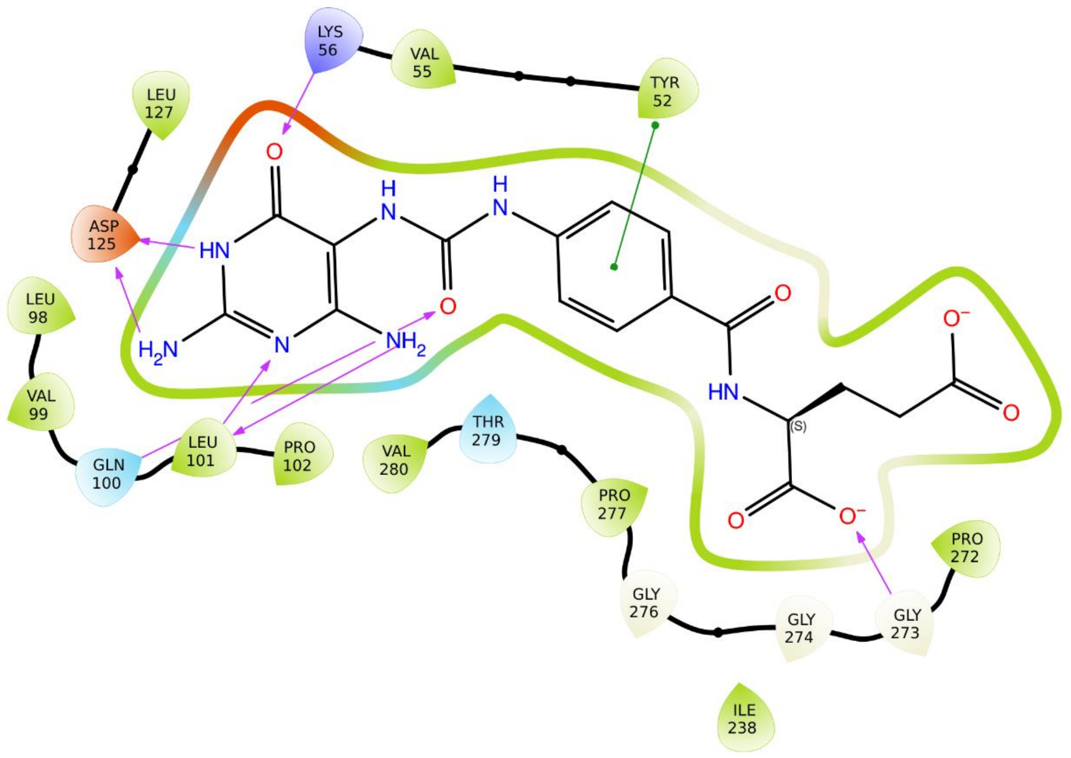

**Figure S5.** 2D Protein-ligand interaction diagram from the docking pose of MTHFD1 – compound 3.

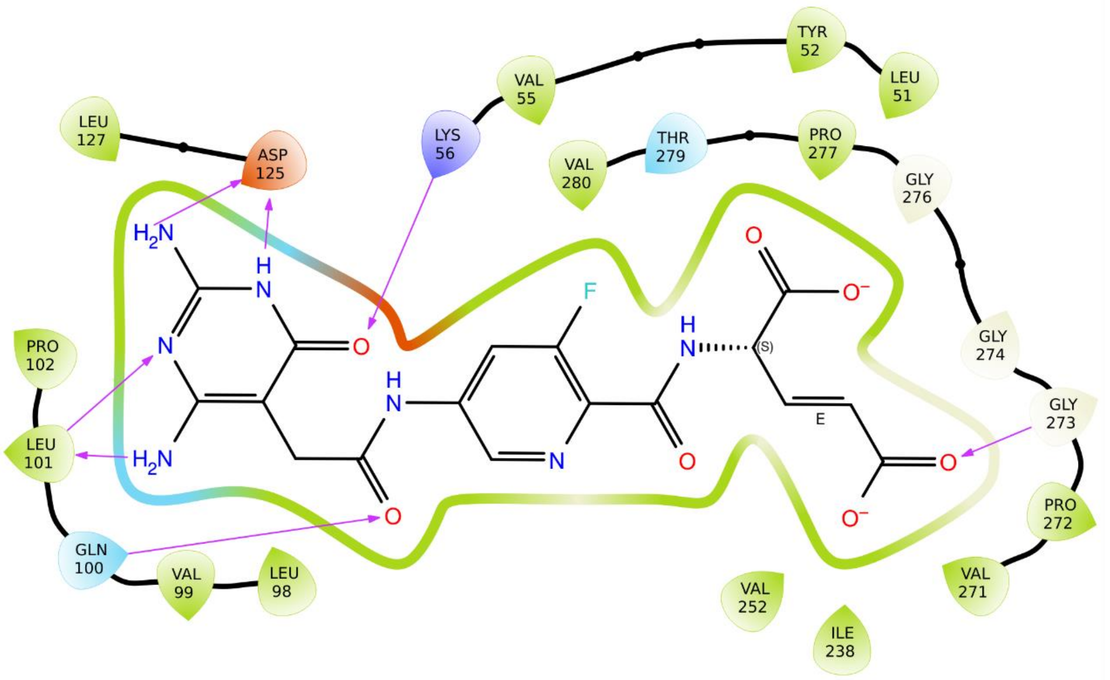


**Figure S6.** RMSD analysis of the MTHFD1 – compound **1** complex from the triplicate MD simulations (A) Protein CA (B) Ligand.

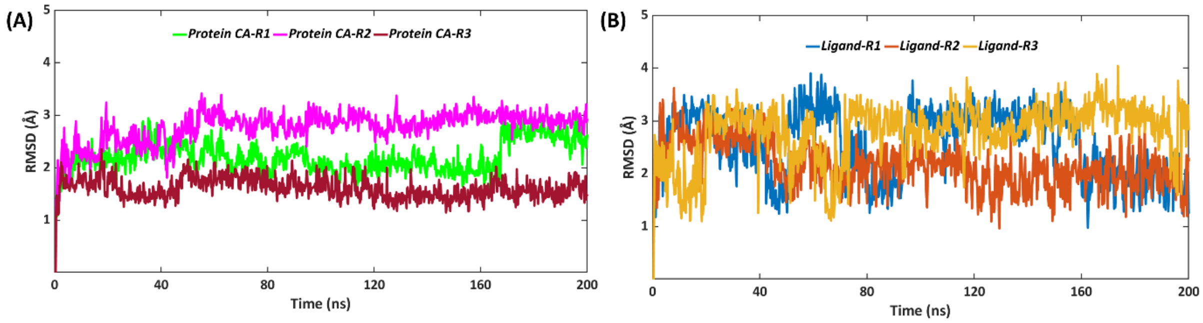


**Table S1.** Average RMSD values for the MTHFD1 – compound **1** complex from the triplicate MD simulations.

| Entry | Average RMSD of Protein CA (Å) | Average RMSD of Ligand (Å) |
|---|---|---|
| **Replica 1** | 2.2 | 2.5 |
| **Replica 2** | 2.7 | 2.1 |
| **Replica 3** | 1.6 | 2.8 |
| **Overall Avg RMSD** | **2.2** | **2.5** |

**Figure S7.** Protein-ligand interaction histogram from the MD simulations of compound **1** in the MTHFD1 binding site. (H-bonds are shown in green, salt-bridge interactions are shown in pink, and lipophilic contacts are shown in grey).

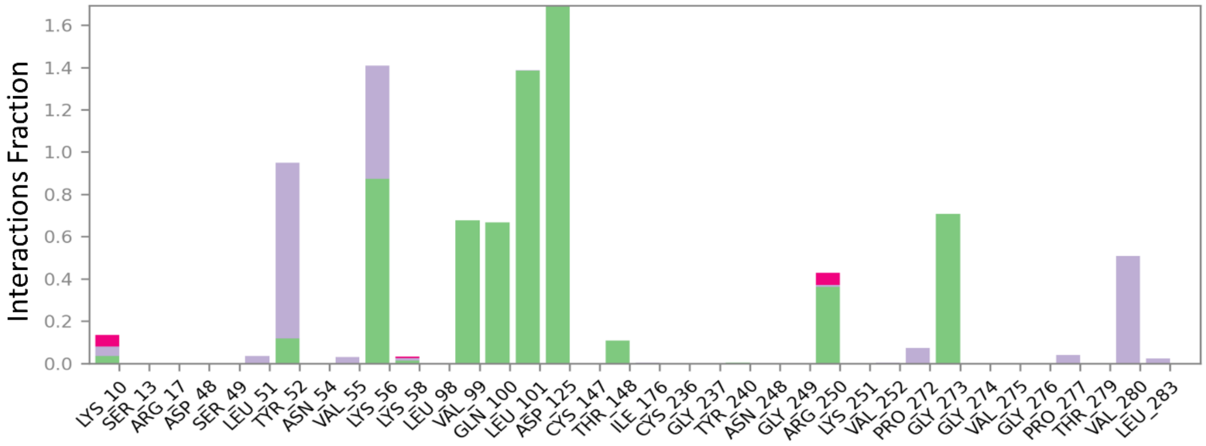


**Figure S8.** RMSD analysis of the MTHFD1 – compound **1** complex during the 200ns simulation. Protein α-carbons are shown in pink while the inhibitor is hidden.

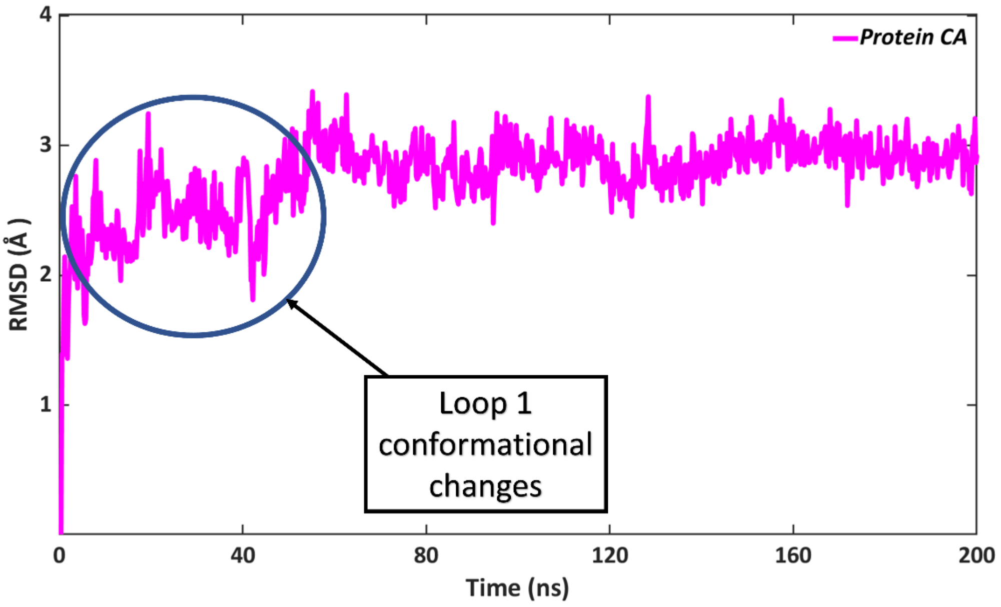

**Figure S9.** MD structure of the MTHFD1 – compound **1** complex at 15th ns superposed with the X-ray structure of MTHFD1 without the substrate site inhibitor (PDB code: 6ECR). MTHFD1-compound **1** complex: protein ribbons in pink, loop 1 in dark blue and compound **1** in orange. MTHFD1 without the inhibitor: protein ribbons in cyan and loop 1 in green.

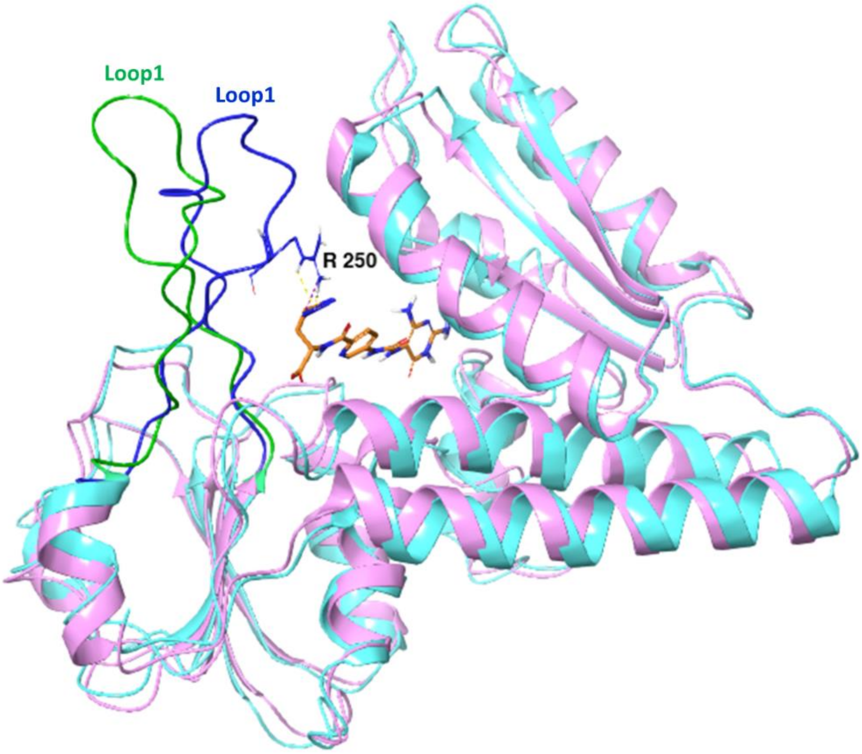


**Figure S10.** RMSD analysis of the MTHFD1 – compound **2** complex from the triplicate MD simulations (A) Protein CA (B) Ligand.

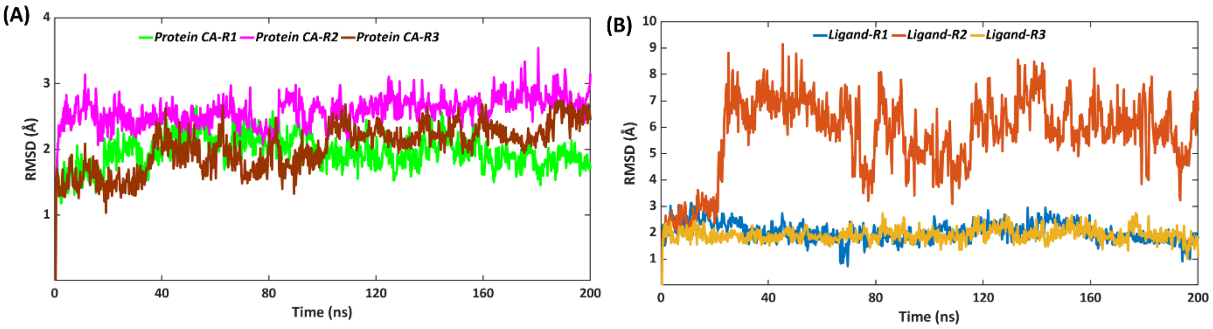

**Table S2.** Average RMSD values for the MTHFD1 – compound **2** complex from the triplicate MD simulations.

| Entry | Average RMSD of Protein CA (Å) | Average RMSD of Ligand (Å) |
|---|---|---|
| **Replica 1** | 1.9 | 2.0 |
| **Replica 2** | 2.6 | 5.7 |
| **Replica 3** | 2.0 | 1.9 |
| **Overall Avg RMSD** | **2.2** | **3.2** |

**Figure S11.** MD snapshots of the MTHFD1 – compound **2** complex at **(A)** 0 ns. **(B)** 41 ns: H-bond interaction with Tyr240 **(C)** 80 ns: H-bond/salt-bridge interaction with Arg250. MTHFD1 protein ribbons are shown in pink, loop 1 in dark blue and compound **2** in green.

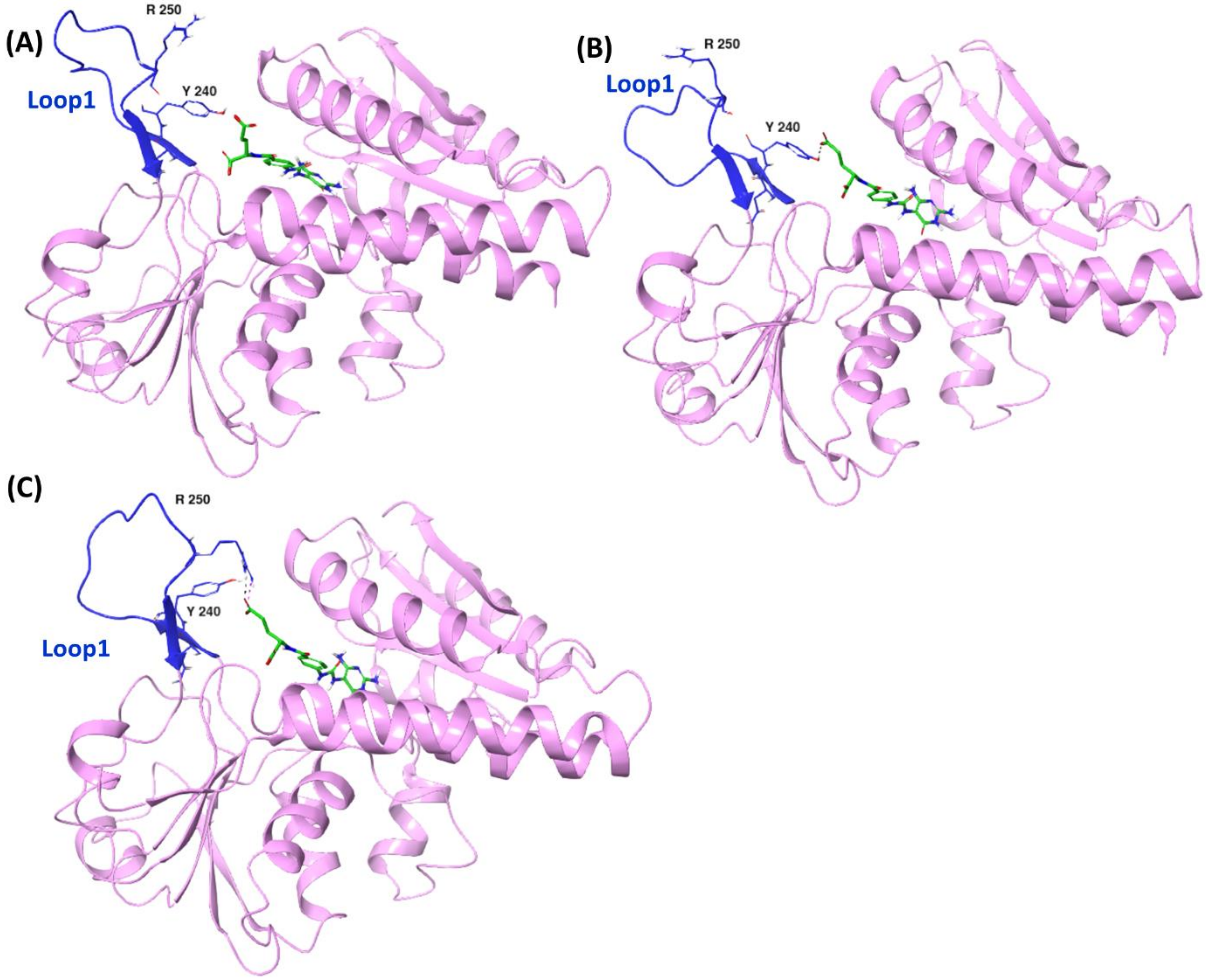

**Figure S12.** Protein-ligand interaction histogram from the MD simulations of compound **2** in the MTHFD1 binding site. (H-bonds are shown in green, salt-bridge interactions are shown in pink, and lipophilic contacts are shown in grey).

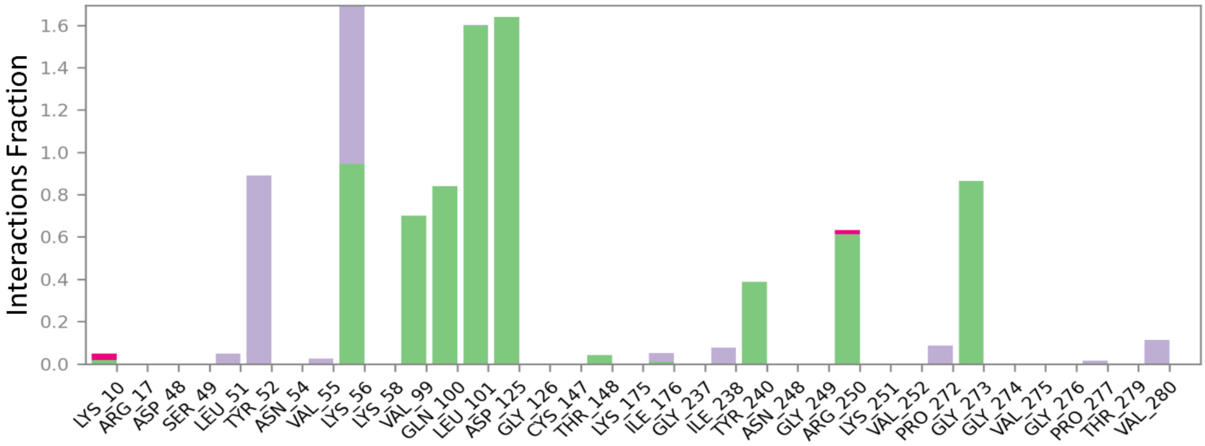


**Figure S13.** RMSD analysis of the MTHFD1 – compound **3** complex from the triplicate MD simulations (A) Protein CA (B) Ligand.

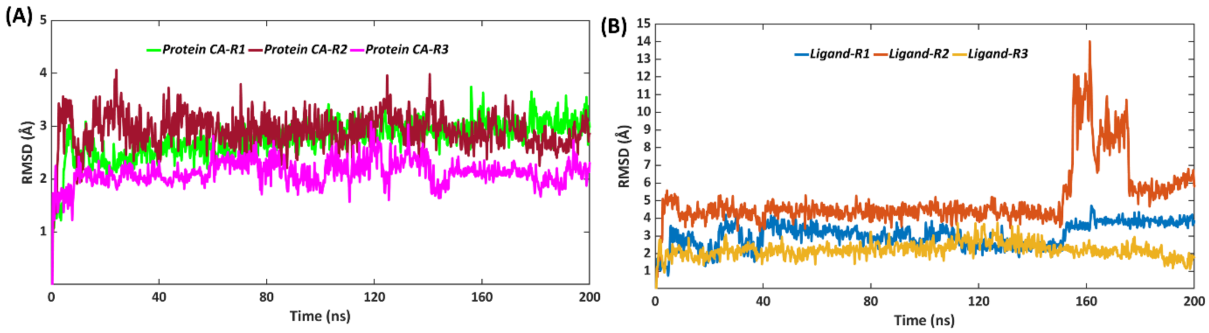


**Table S3.** Average RMSD values for the MTHFD1 – compound **3** complex from the triplicate MD simulations.

| **Entry** | **Average RMSD of Protein CA (Å)** | **Average RMSD of Ligand (Å)** |
|---|---|---|
| **Replica 1** | 2.7 | 3.0 |
| **Replica 2** | 2.9 | 5.0 |
| **Replica 3** | 2.1 | 2.2 |
| **Overall Avg RMSD** | **2.6** | **3.4** |

**Figure S14.** Protein-ligand interaction histogram from the MD simulations of compound **3** in the MTHFD1 binding site. (H-bonds are shown in green, salt-bridge interactions are shown in pink, and lipophilic contacts are shown in grey).

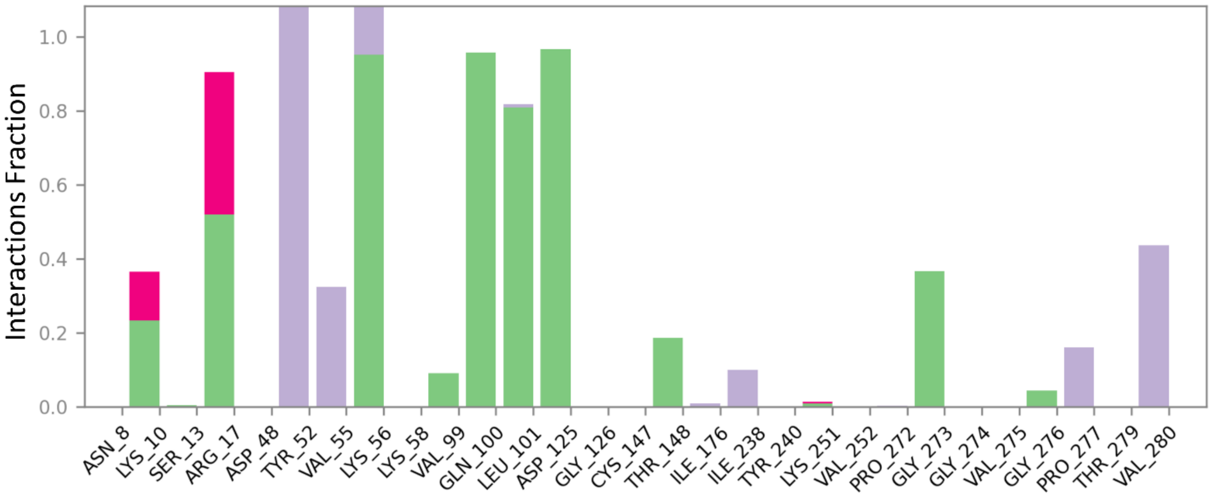


**Figure S15.** RMSD analysis of the MTHFD2 – compound **1** complex from the triplicate MD simulations (A) Protein CA (B) Ligand.

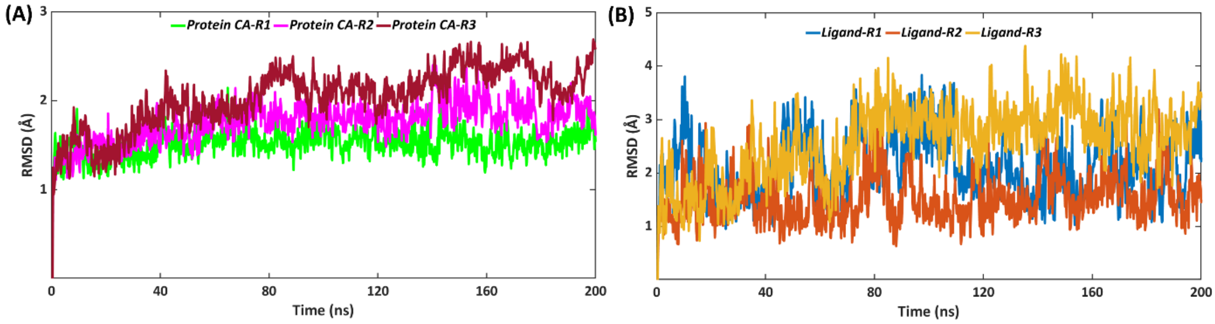


**Table S4.** Average RMSD values for the MTHFD2 – compound **1** complex from the triplicate MD simulations.

| Entry | Average RMSD of Protein CA (Å) | Average RMSD of Ligand (Å) |
|---|---|---|
| **Replica 1** | 1.5 | 2.1 |
| **Replica 2** | 1.7 | 1.5 |
| **Replica 3** | 2.0 | 2.6 |
| **Overall Avg RMSD** | **1.7** | **2.0** |

**Figure S16. (A)** Co-crystallized pose of compound **1** in the MTHFD2 binding site (ligand in orange, protein residues in blue, PDB code: 6S4A). **(B)** Co-crystallized pose of compound **2** in the MTHFD2 binding site (ligand in green, protein residues in blue, PDB code: 6S4E). **(C)** Co-crystallized pose of compound **3** in the MTHFD2 binding site (ligand in red, protein residues in blue, PDB code: 6S4F).

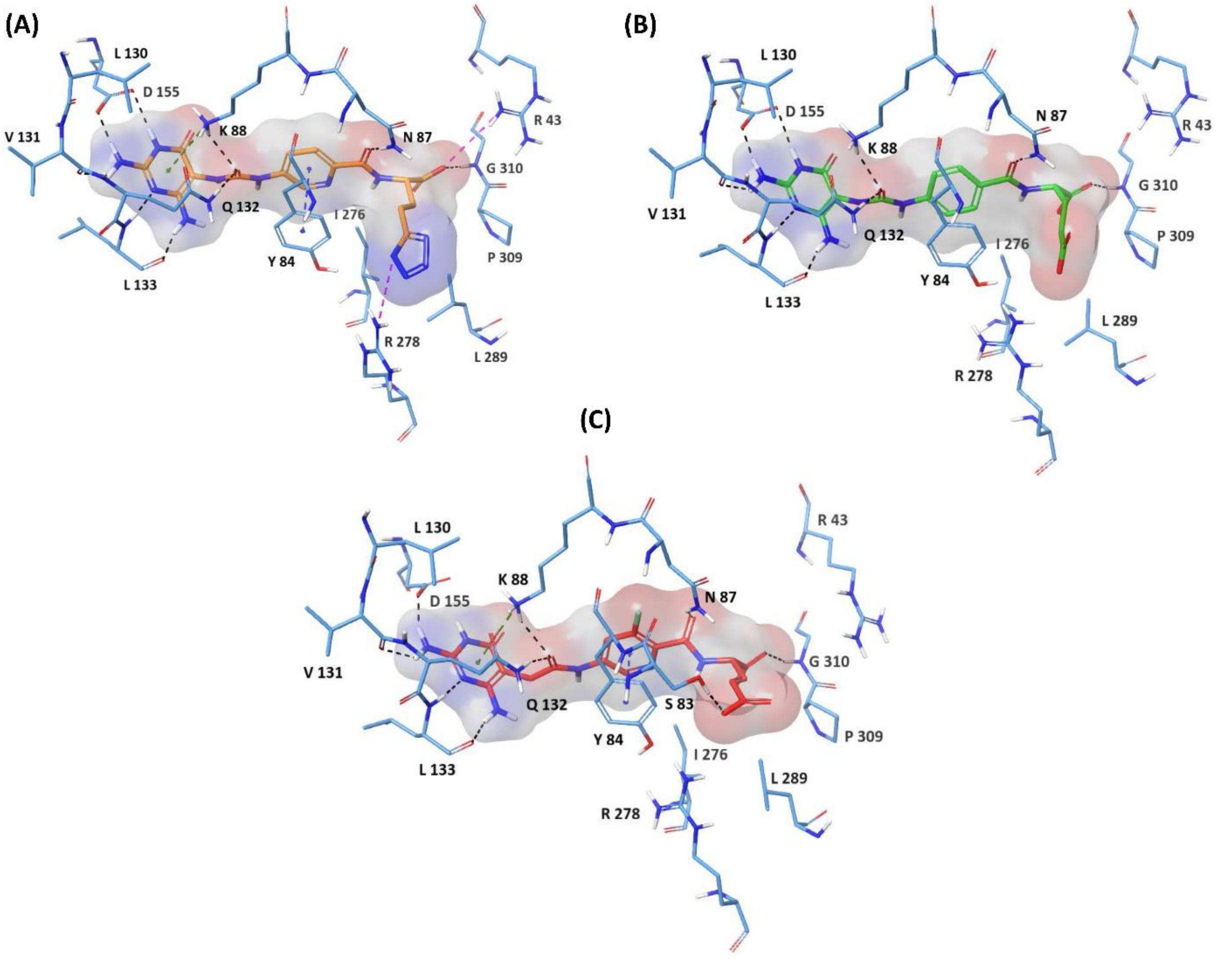

**Figure S17.** 2D Protein-ligand interaction diagram from the co-crystallized pose of MTHFD2 – compound 1.

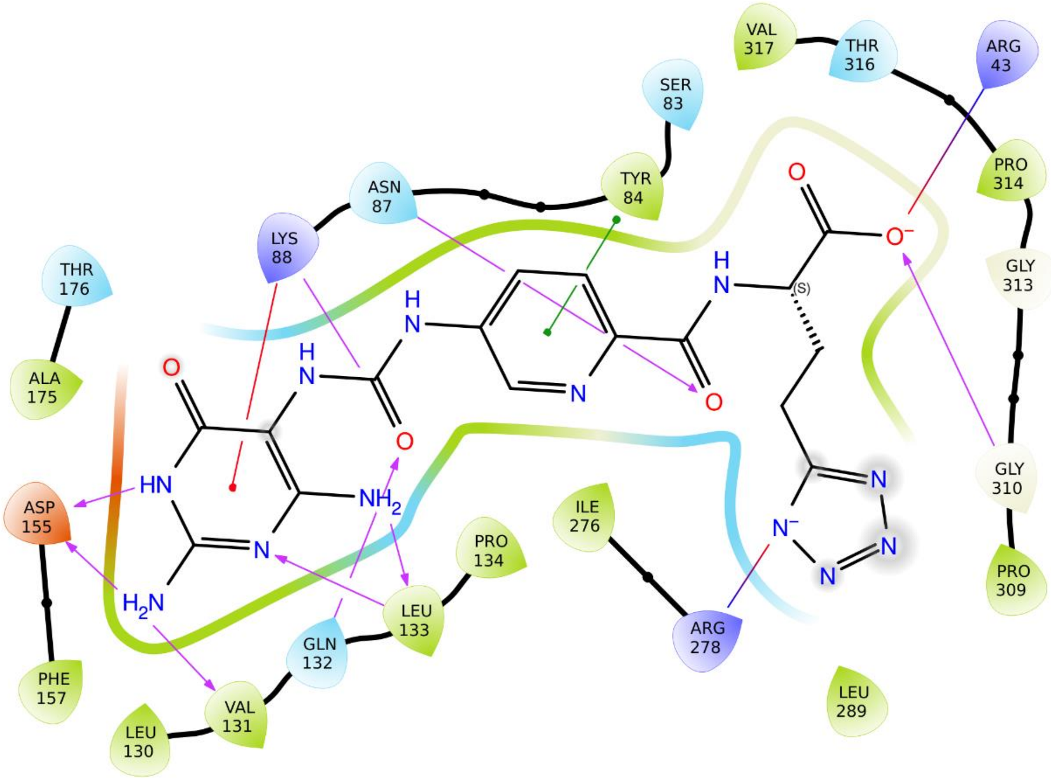


**Figure S18.** 2D Protein-ligand interaction diagram from the co-crystallized pose of MTHFD2 – compound 2.

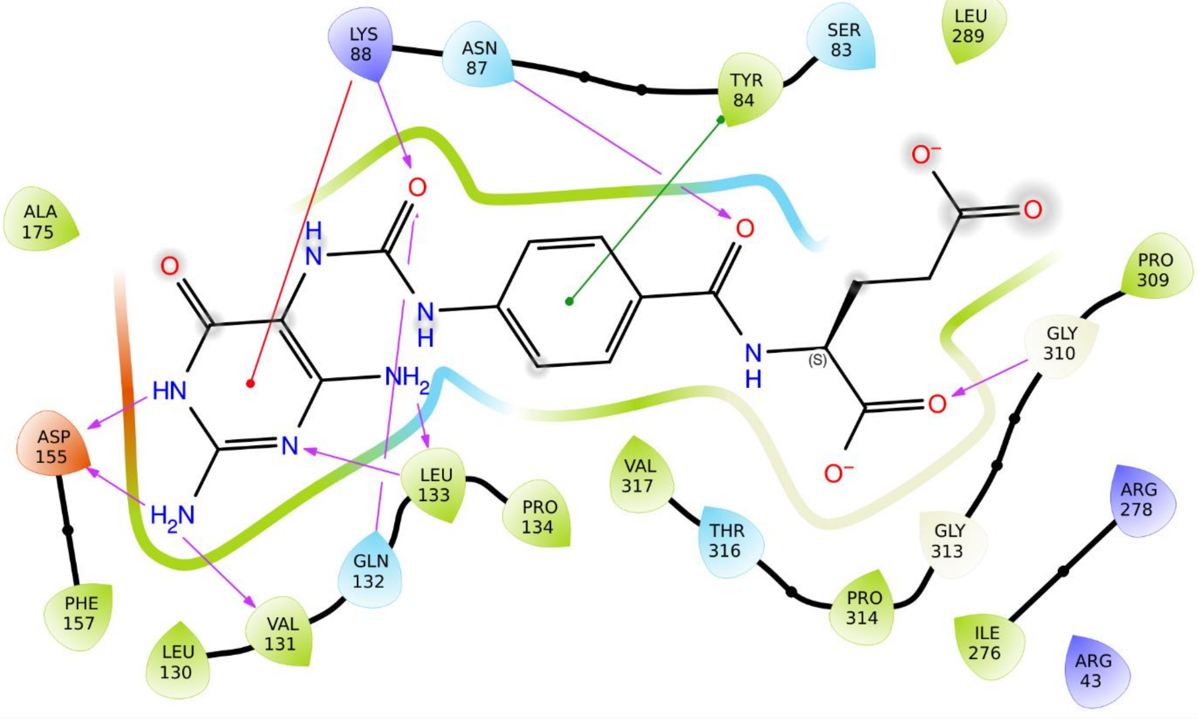

**Figure S19.** 2D Protein-ligand interaction diagram from the co-crystallized pose of MTHFD2 – compound 3.

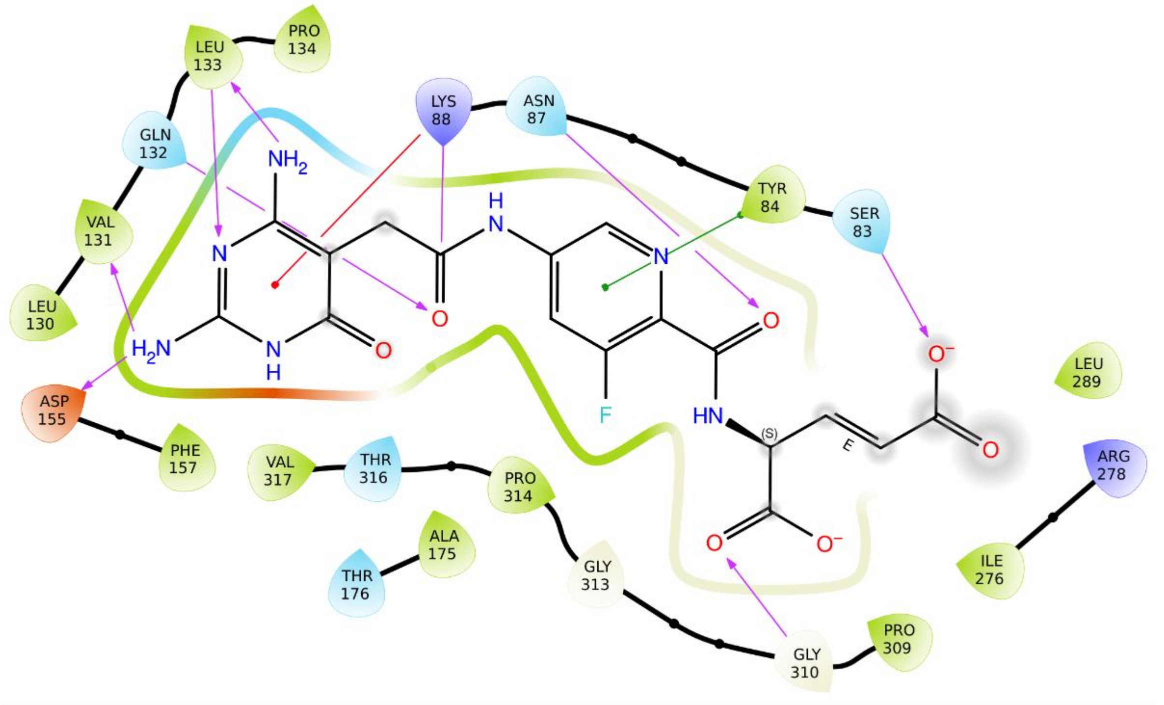


**Figure S20.** Protein-ligand interaction histogram from the MD simulations of compound **1** in the MTHFD2 binding site. (H-bonds are shown in green, salt-bridge interactions are shown in pink, and lipophilic contacts are shown in grey).

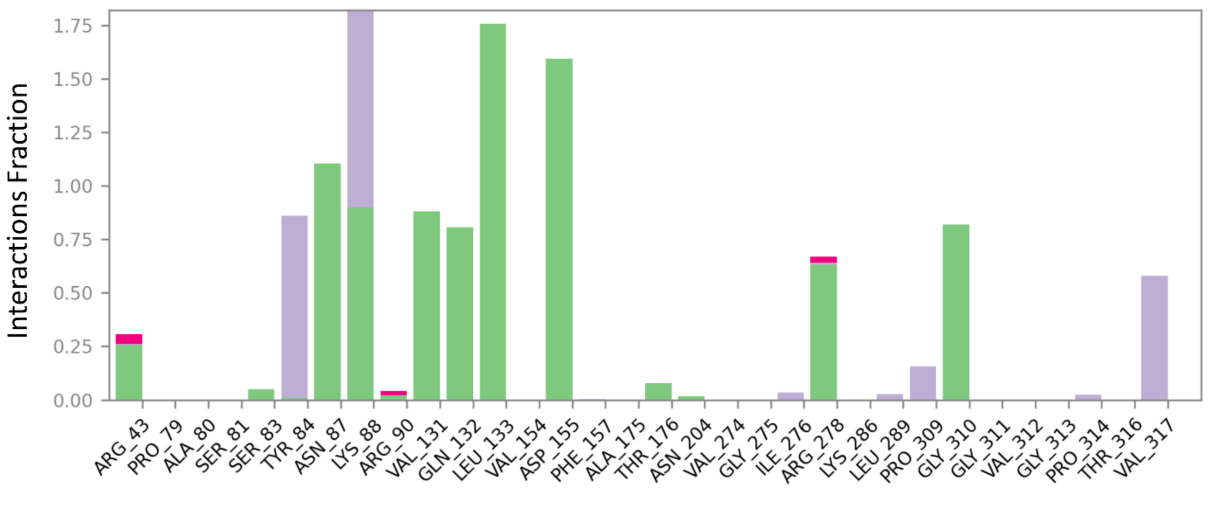

**Figure S21.** RMSD analysis of the MTHFD2 – compound **2** complex from the triplicate MD simulations (A) Protein CA (B) Ligand.

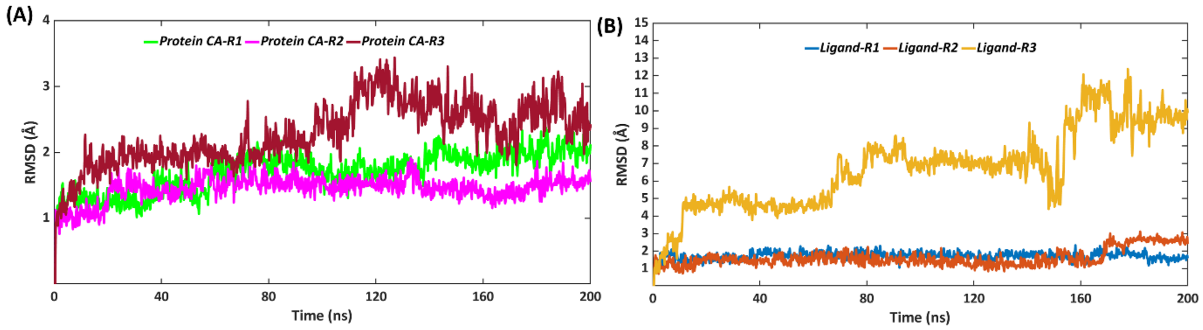


**Table S5.** Average RMSD values for the MTHFD2 – compound **2** complex from the triplicate MD simulations.

| Entry | Average RMSD of Protein CA (Å) | Average RMSD of Ligand (Å) |
|---|---|---|
| **Replica 1** | 1.7 | 1.7 |
| **Replica 2** | 1.4 | 1.6 |
| **Replica 3** | 2.9 | 6.7 |
| **Overall Avg RMSD** | **2** | **3.3** |

**Figure S22.** Protein-ligand interaction histogram from the MD simulations of compound **2** in the MTHFD2 binding site. (H-bonds are shown in green, salt-bridge interactions are shown in pink, and lipophilic contacts are shown in grey).

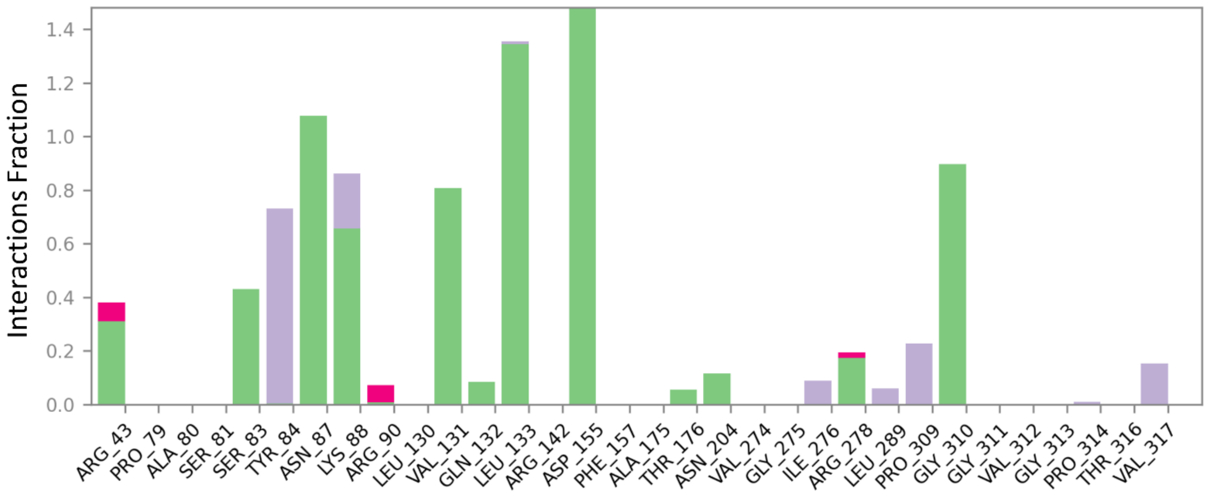

**Figure S23.** RMSD analysis of the MTHFD2 – compound **3** complex from the triplicate MD simulations (A) Protein CA (B) Ligand.

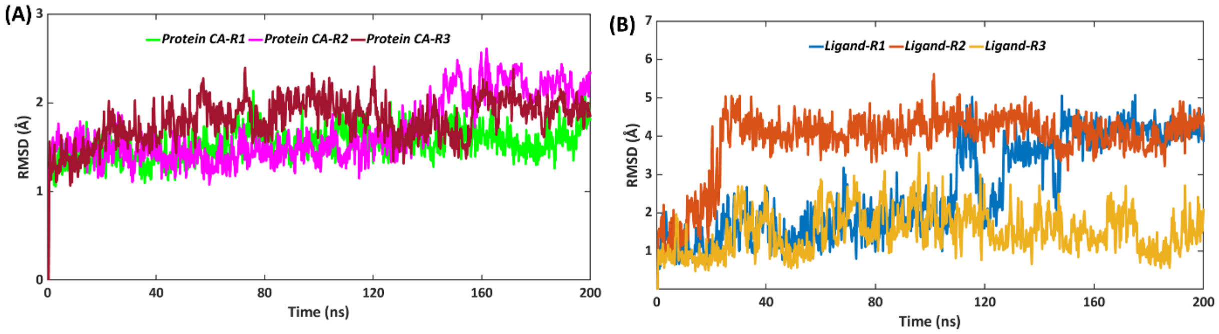


**Table S6.** Average RMSD values for the MTHFD2 – compound **3** complex from the triplicate MD simulations.

| Entry | Average RMSD of Protein CA (Å) | Average RMSD of Ligand (Å) |
|---|---|---|
| **Replica 1** | 1.5 | 2.6 |
| **Replica 2** | 1.6 | 3.9 |
| **Replica 3** | 1.8 | 1.5 |
| **Overall Avg RMSD** | **1.6** | **2.7** |

**Figure S24.** Protein-ligand interaction histogram from the MD simulations of compound **3** in the MTHFD2 binding site. (H-bonds are shown in green, salt-bridge interactions are shown in pink, and lipophilic contacts are shown in grey).

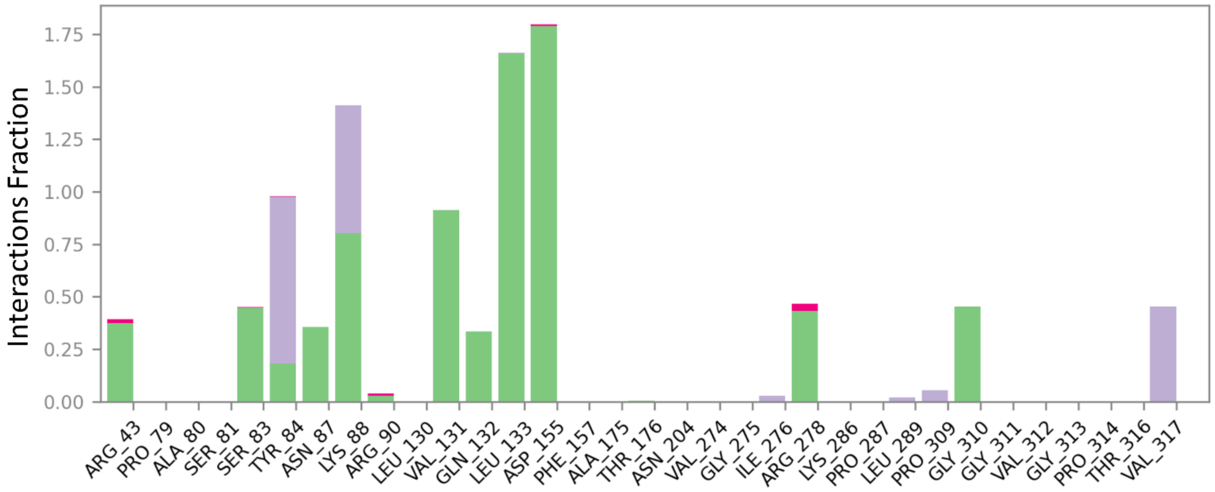

**Figure S25. (A)** Docking pose of compound **1** in the MTHFD2L binding site (ligand in orange, protein residues in grey). **(B)** Crystallographic pose of compound **2** in the MTHFD2L binding site (PDB code: 7QEI, ligand in green, protein residues in grey). **(C)** Docking pose of compound **3** in the MTHFD2L binding site (ligand in red, protein residues in grey).

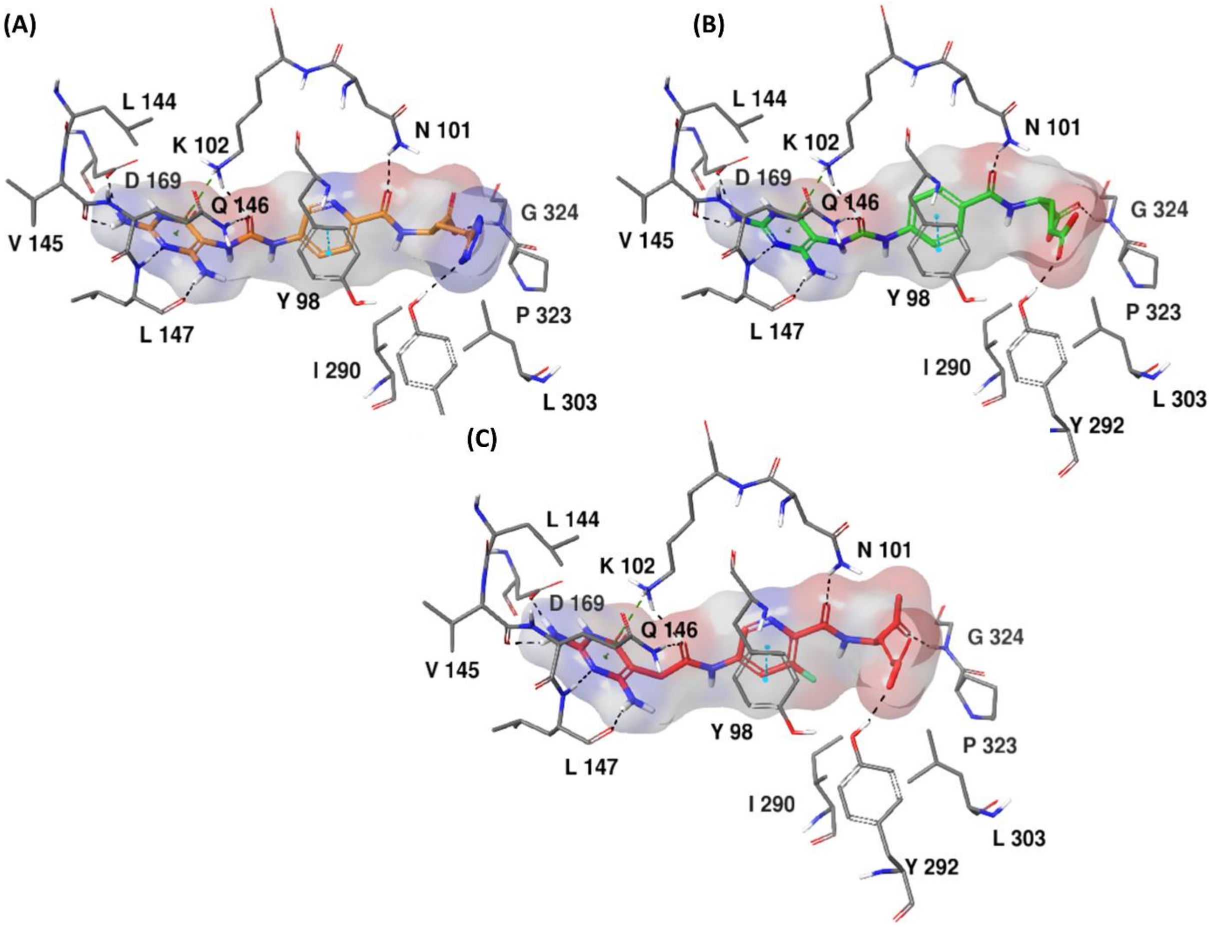


**Figure S26.** 2D Protein-ligand interaction diagram from the docking pose of MTHFD2L – compound 1.

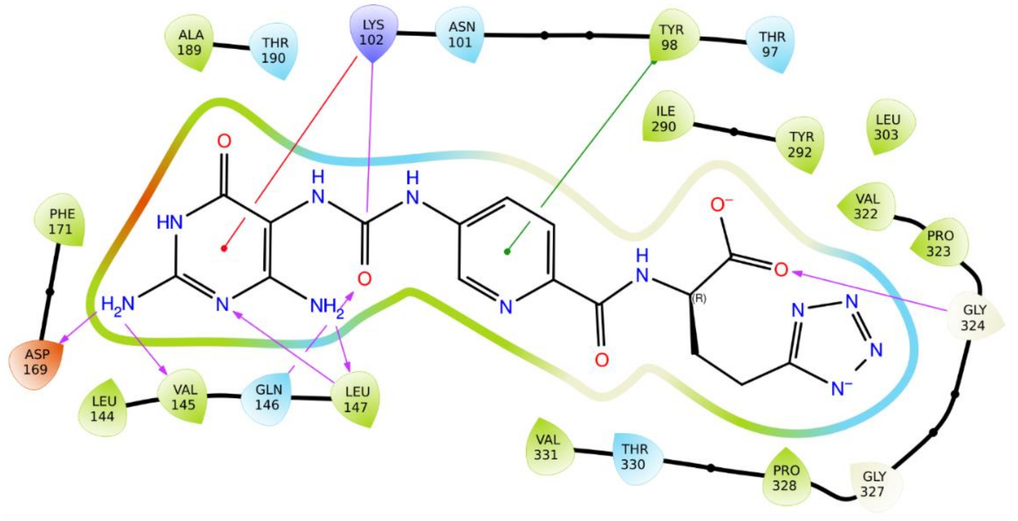

**Figure S27.** 2D Protein-ligand interaction diagram from the co-crystallized pose of MTHFD2L – compound 2.

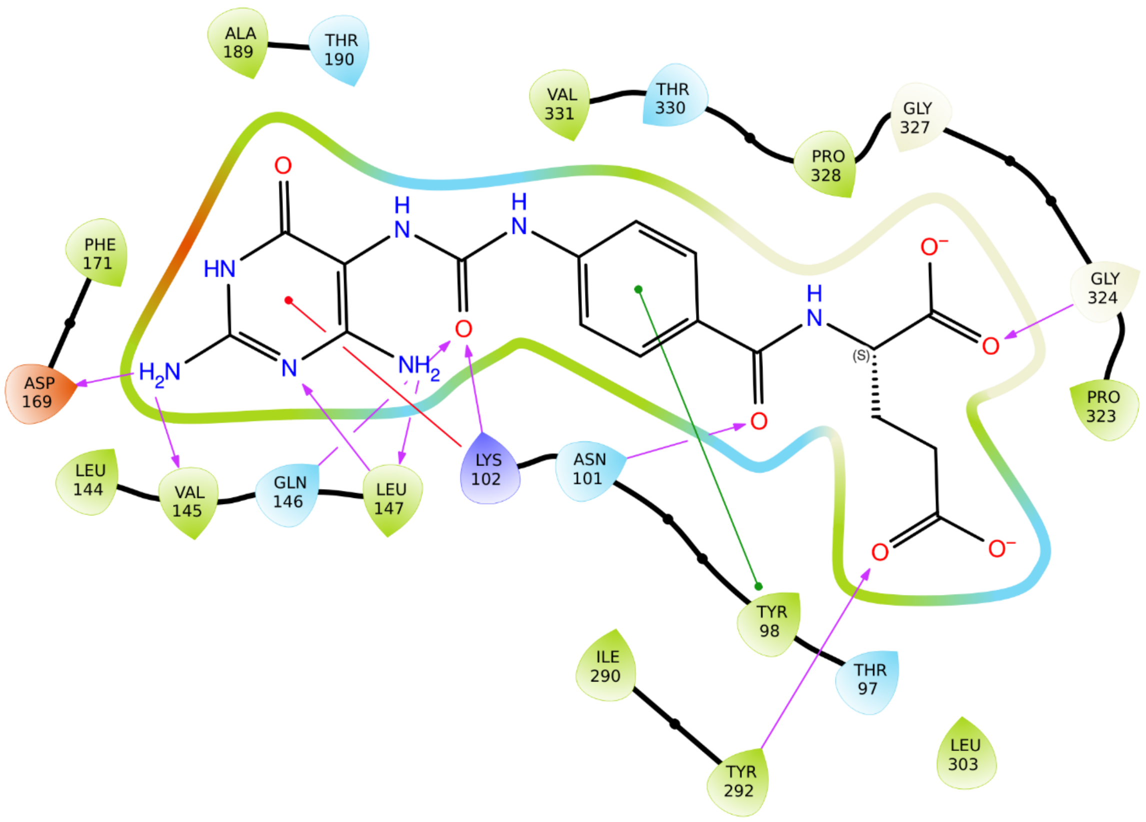


**Figure S28.** 2D Protein-ligand interaction diagram from the docking pose of MTHFD2L – compound 3.

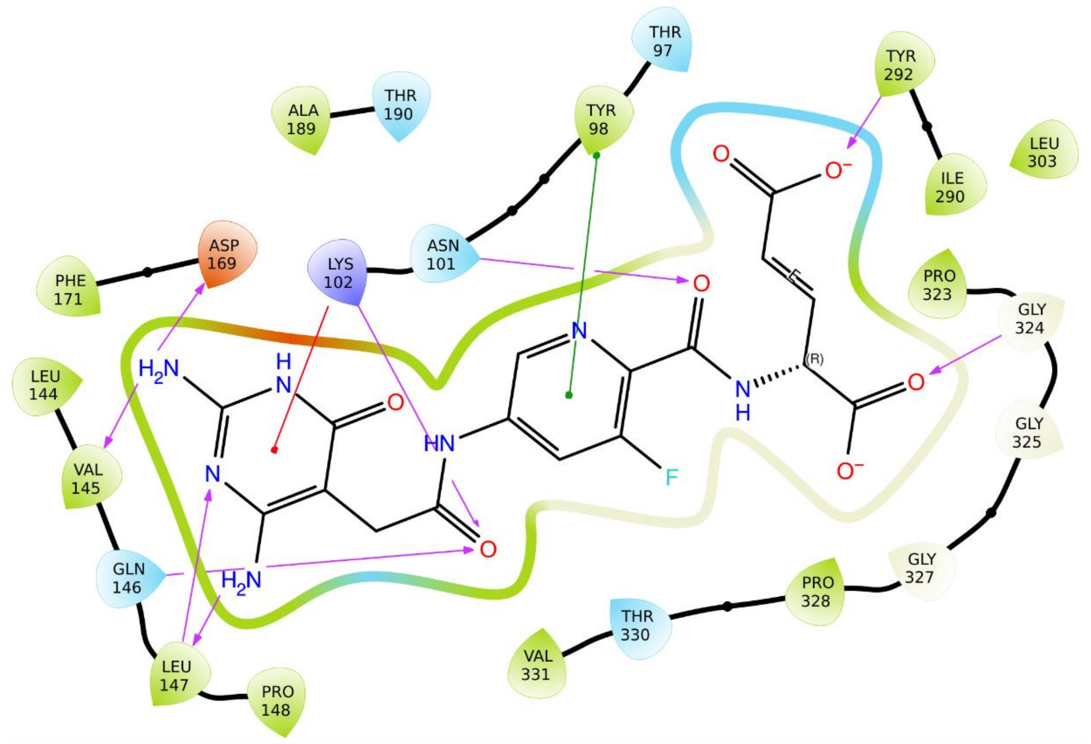

**Figure S29.** RMSD analysis of the MTHFD2L – compound **1** complex from the triplicate MD simulations (A) Protein CA (B) Ligand.

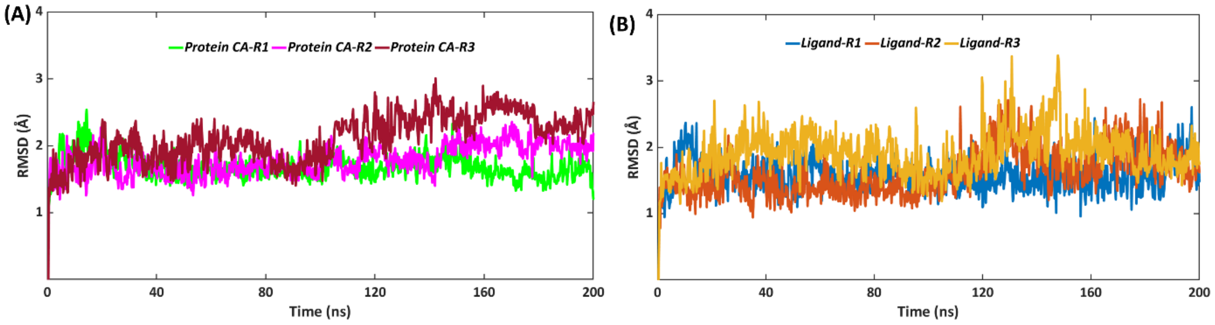


**Table S7.** Average RMSD values for the MTHFD2L – compound **1** complex from the triplicate MD simulations.

| Entry | Average RMSD of Protein CA (Å) | Average RMSD of Ligand (Å) |
|---|---|---|
| **Replica 1** | 1.7 | 1.6 |
| **Replica 2** | 1.8 | 1.6 |
| **Replica 3** | 2.1 | 1.9 |
| **Overall Avg RMSD** | **1.9** | **1.7** |

**Figure S30.** Protein-ligand interaction histogram from the MD simulations of compound **1** in the MTHFD2L binding site. (H-bonds are shown in green, salt-bridge interactions are shown in pink, and lipophilic contacts are shown in grey).

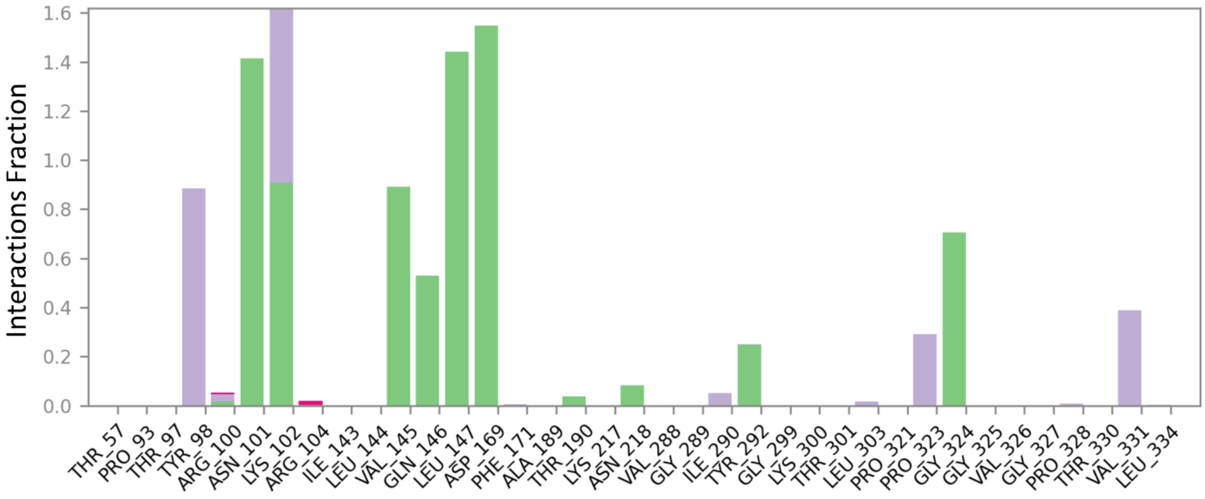

**Figure S31.** RMSD analysis of the MTHFD2L – compound **2** complex from the triplicate MD simulations (A) Protein CA (B) Ligand.

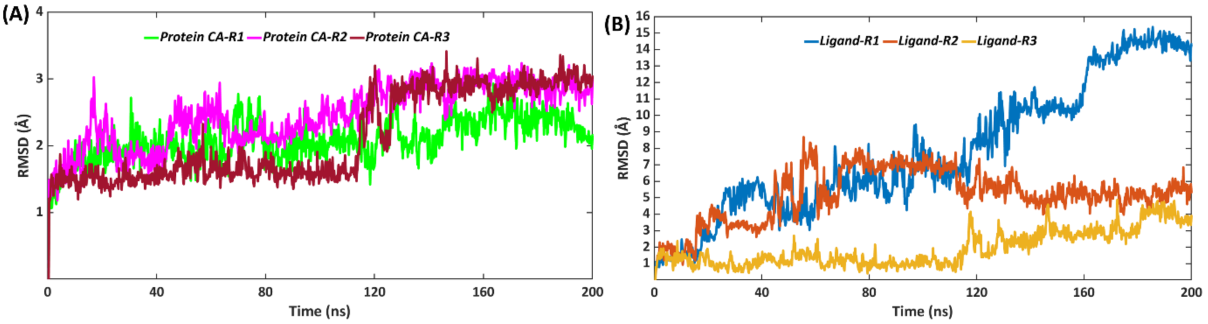


**Table S8.** Average RMSD values for the MTHFD2L – compound **2** complex from the triplicate MD simulations.

| Entry | Average RMSD of Protein CA (Å) | Average RMSD of Ligand (Å) |
|---|---|---|
| **Replica 1** | 2.0 | 7.9 |
| **Replica 2** | 2.5 | 5.2 |
| **Replica 3** | 2.1 | 1.9 |
| **Overall Avg RMSD** | **2.2** | **5.0** |

**Figure S32.** MD snapshots of the MTHFD2L – compound **2** complex at **(A)** 104 ns. **(B)** 120 ns. MTHFD2L protein ribbons are shown in grey, loop 1 in dark blue, α-helix 1 in magenta and compound **2** in green.

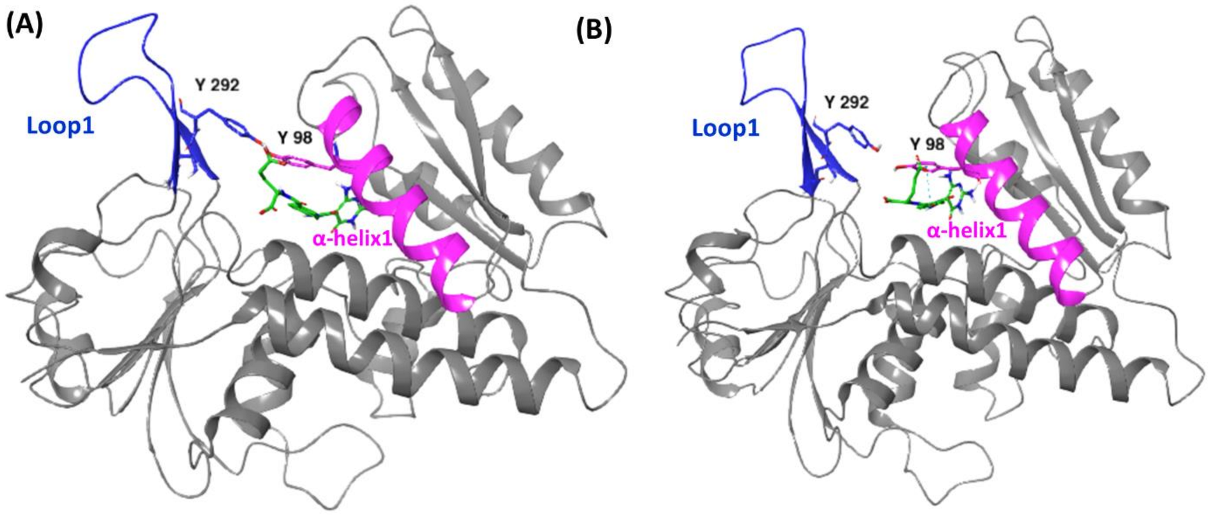

**Figure S33** Protein-ligand interaction histogram from the MD simulations of compound **2** in the MTHFD2L binding site. (H-bonds are shown in green, salt-bridge interactions are shown in pink, and lipophilic contacts are shown in grey).

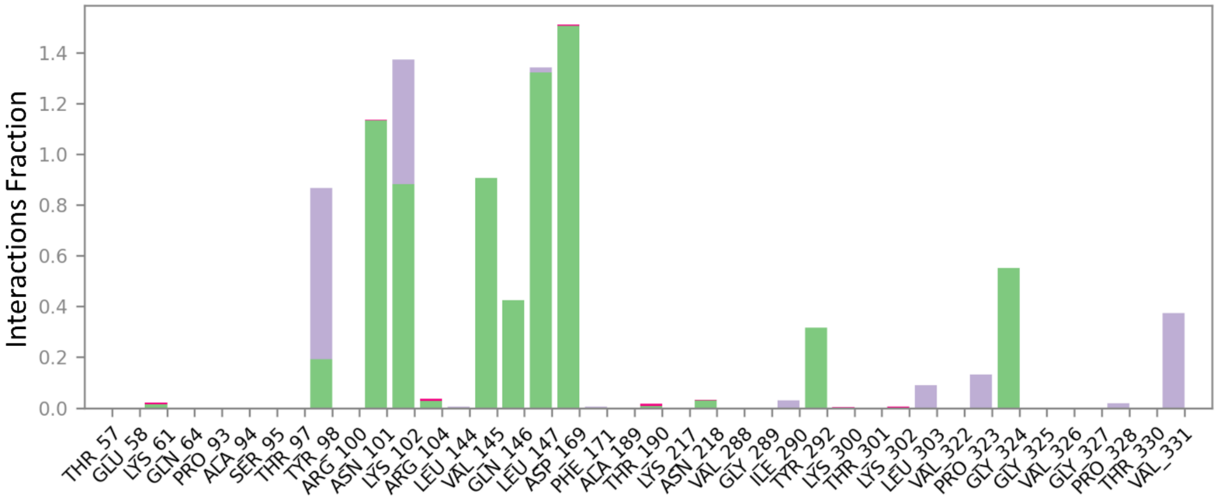


**Figure S34.** RMSD analysis of the MTHFD2L – compound **3** complex from the triplicate MD simulations (A) Protein CA (B) Ligand.

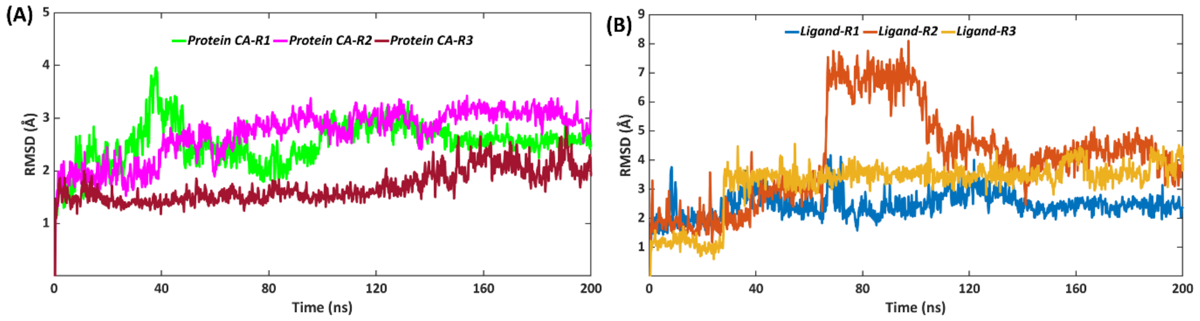


**Table S9.** Average RMSD values for the MTHFD2L – compound **3** complex from the triplicate MD simulations.

| Entry | Average RMSD of Protein CA (Å) | Average RMSD of Ligand (Å) |
|---|---|---|
| **Replica 1** | 2.5 | 2.5 |
| **Replica 2** | 2.7 | 4.0 |
| **Replica 3** | 1.7 | 3.2 |
| **Overall Avg RMSD** | **2.3** | **3.2** |

**Figure S35.** Protein-ligand interaction histogram from the MD simulations of compound **3** in the MTHFD2L binding site. (H-bonds are shown in green, salt-bridge interactions are shown in pink, and lipophilic contacts are shown in grey).

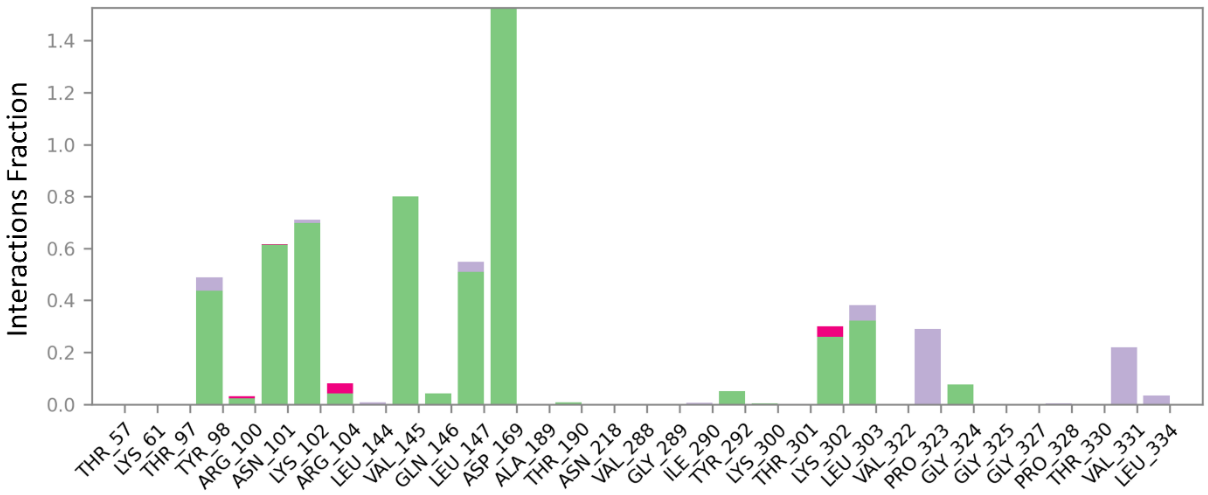

Supplement: Supplementary file 1 — Supplementary Information. [file 41598_2024_71879_MOESM1_ESM.pdf]
